# Enhancement of Magnetic Shielding Based on Low-Noise Materials, Magnetization Control, and Active Compensation: A Review

**DOI:** 10.3390/ma17225469

**Published:** 2024-11-08

**Authors:** Yijin Liu, Jianzhi Yang, Fuzhi Cao, Xu Zhang, Shiqiang Zheng

**Affiliations:** 1 Key Laboratory of Ultra-Weak Magnetic Field Measurement Technology, Ministry of Education, School of Instrumentation and Optoelectronic Engineering, Beihang University, Beijing 100191, China; 2 Zhejiang Provincial Key Laboratory of Ultra-Weak Magnetic-Field Space and Applied Technology, Hangzhou Innovation Institute, Beihang University, Hangzhou 310051, China

**Keywords:** magnetic shield, low-noise material, degaussing, magnetic shaking, active compensation

## Abstract

Magnetic-shielding technologies play a crucial role in the field of ultra-sensitive physical measurement, medical imaging, quantum sensing, etc. With the increasing demand for the accuracy of magnetic measurement, the performance requirements of magnetic-shielding devices are also higher, such as the extremely weak magnetic field, gradient, and low-frequency noise. However, the conventional method to improve the shielding performance by adding layers of materials is restricted by complex construction and inherent materials noise. This paper provides a comprehensive review about the enhancement of magnetic shielding in three aspects, including low-noise materials, magnetization control, and active compensation. The generation theorem and theoretical calculation of materials magnetic noise is summarized first, focusing on the development of spinel ferrites, amorphous, and nanocrystalline. Next, the principles and applications of two magnetization control methods, degaussing and magnetic shaking, are introduced. In the review of the active magnetic compensation system, the forward and inverse design methods of coil and the calculation method of the coupling effect under the ferromagnetic boundary of magnetic shield are explained in detail, and their applications, especially in magnetocardiography (MCG) and magnetoencephalogram (MEG), are also mainly described. In conclusion, the unresolved challenges of different enhancement methods in materials preparation, optimization of practical implementation, and future applications are proposed, which provide comprehensive and instructive references for corresponding research.

## 1. Introduction

In recent decades, with the rapid development of quantum theory and the increasing maturity of quantum sensors, ultra-sensitive measurements of physical quantities, such as magnetic field [1,2], inertial navigation [3,4], gravity [5,6], and time [7,8], have been achieved through the quantum manipulation of atoms, electrons, and nucleons. Spin-Exchange Relaxation-Free (SERF) atomic magnetometer [9] has surpassed Superconducting Quantum Interference Device (SQUID) [10] as the new quantum magnetic sensor, which improves the sensitivity of magnetic measurement from the order of fT to aT theoretically [11]. With its advantages of high sensitivity and miniaturization, SERF atomic magnetometers play a key role in the field of extremely weak biological magnetic field measurement represented by magnetocardiography (MCG) [12,13] and magnetoencephalogram (MEG) [14,15]. The nearly zero-field environment is a necessary condition to realize the high sensitivity of SERF atomic magnetometers and meanwhile protects the extremely weak magnetic signal from the interference of external complex magnetic environment [16,17]. Therefore, a low-noise and high-performance magnetic-shielding device is essential for the above studies.

Magnetic-shielding technology began to develop in the 1960s, gradually matured in the 1990s, and can be widely used in a variety of disciplines with weak magnetic fields as the research background. The magnetic-shielding devices mainly include two types: magnetically shielded room (MSR) [18,19,20] and cylindrical magnetic shield [21,22,23], which are composed of high-permeability layers and high-conductive layers, realizing the shielding functions based on the flux shunting and eddying effects, respectively. The shielding performance is determined by the structure of the magnetic-shielding device as well as the magnetic properties of the shielding materials, which can be significantly enhanced by increasing the number of the layers and the amount of the shielding materials [24]. However, the increased construction period and cost limit the further application, while the added layers and complex structure result in the extra magnetic noise [25] and inhomogeneous distribution of residual field [26], respectively. It is necessary to summarize the principle and characteristics of different shielding enhancements and adopt corresponding methods according to actual applications and requirements.

Magnetic-shielding devices with multi layers of traditional soft magnetic materials and conductive materials realize great shielding effects for most environmental magnetic fields. However, in the field of ultra-high precision measurement, such as dark matter particle explore [27,28] and electric dipole moment measurement [29,30], the inherent magnetic properties of shielding devices become the main restrictions for magnetic sensitive physical measurements. Under the circumstance of extremely weak magnets, the magnetic noise caused by eddy current and magnetic domain fluctuations within the shielding materials significantly affects the magnetic response sensitivity, especially for atomic devices [31,32]. On the basis of magnetic-shielding devices, the interior layer of low-noise materials can further shield the intrinsic noise of the high-permeability layers and high-conductive layers. Therefore, the composite shielding structure with the innermost layer made of low-noise materials such as ferrites and nanocrystalline provide a more ideal environment with low magnetic noise [33].

For the measurement of biological field such as MCG [34] and MEG [35], magnetic noise from shielding materials is not the dominant factor due to its lower magnitude than the tested signal [36]. The method of magnetization control and active compensation can effectively enhance the shielding performance without additional shielding layers [37,38]. Degaussing, also called magnetic equilibration, as the most common method of magnetization control, can eliminate the extra remanence of the high-permeability shielding material due to transportation, installation, and other processes, which reduces the residual field and field gradient inside magnetic shield [26,39]. At present, the static shielding performance after magnetic equilibrium is still far from the theoretical limit, and further analysis and optimization of this method are necessary [40]. Magnetic shaking, as another magnetization control method, can suppress the loss during the magnetization process and improve the shielding ability for the low-frequency magnetic field by introducing an alternating current (AC) field into shielding layers [41,42]. There are still some problems in the practical application of magnetic shaking, such as the low efficiency and additional noise sources.

Compared with the method of magnetization control, the active magnetic compensation based on the coil system can directly reduce the residual field and suppress the magnetic noise by generating the field in a contrary direction. Firstly, the compensation effectiveness is determined by the performance of the compensation coils, so a series of design methods are proposed, which can be mainly divided into forward [43] and inverse methods [44,45] according to the principle. The forward methods are more suitable for the design of regular-shape coils with high uniformity of magnetic field or field gradient, such as Helmholtz coil and Maxwell coil. For the limited space in the magnetic-shielding device, the irregular-shape coil configurations can be designed by the inverse methods on the surface of plane, cylinder, sphere, or complex shape structure appropriate to the special requirements in the measurements [46]. In addition, the compensation field generated by the coils can be distorted due to the magnetic-shielding layers with high permeability. Therefore, this coupling effect should be further analyzed and calculated in the design process of the coil system based on the theoretical analysis method or image method to avoid the reduction in the compensation effectiveness [47,48].

**Figure 1:**
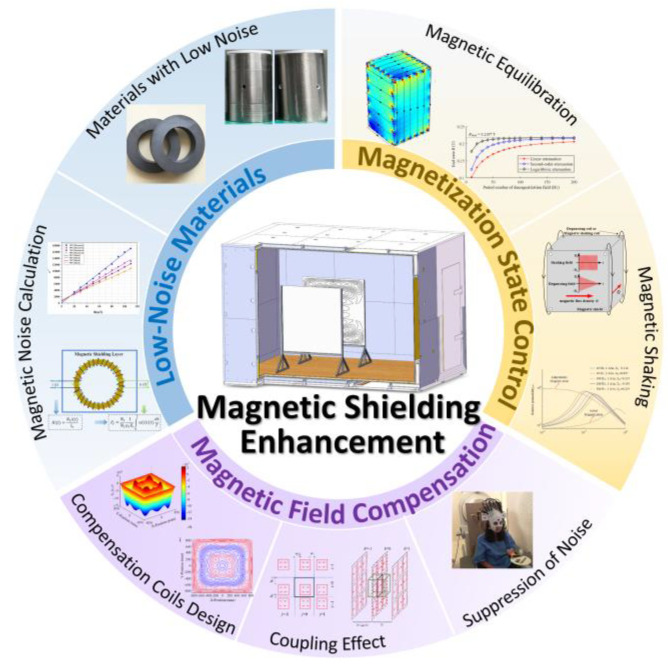
Enhancement methods of magnetic shielding based on low-noise materials, magnetization control, and active compensation.

In this article, the enhancement methods of magnetic shielding based on low-noise materials, magnetization control, and active compensation are reviewed as shown in Figure 1. The subsequent content is organized as follows. In Section 2, the principle and theories on magnetic shielding are summarized. Section 3 focus the calculation method of the material noise, and introduces the low-noise materials, mainly including spinel ferrites, amorphous, and nanocrystalline. And then, the principles and research of magnetic equilibration and magnetic shaking, two common magnetization control methods, are explained and summarized in Section 4. The design methods and applications of compensation coils are illustrated in Section 5. Section 6 concludes this paper and gives an outlook for the future developments on these enhancement methods.

## 2. Principle and Applications of Magnetic Shielding

An internal space with weak magnetic field can be obtained based on magnetic-shielding devices. There are three classifications of magnetic-shielding devices according to different shielding materials and shielding principles [24]: (1) Made of materials with high magnetic permeability, such as Permalloy, the magnetic-shielding devices directly converge magnetic field lines into these materials based on the principle of magnetic flux shunting. By preventing their diffusion into the shielding devices, the shielding against static and low-frequency magnetic fields is achieved. (2) With conducting materials like copper and aluminum, a magnetic field in a contrary direction generated by an eddy current effect can counteract the external environmental field. By adopting a high-sealed and high-conductivity shell, the magnetic-shielding device can fulfill the shielding against electromagnetic interference with high frequency. (3) The Meissner effect of superconducting shields can expulse magnetic fields from the interior of superconductors when cooled below the transition temperature, in the process of which the material losses its resistance to the flow of electrical currents [49,50]. However, commonly used superconductors require extremely low temperatures, typically with liquid helium cooling, incurring high operational costs. Therefore, the applications of superconducting shields are restricted and most magnetic-shielding devices are designed to combine the magnetic circuit-shunting effect of high-permeability materials and the eddy current effect of high-conductivity materials. 

For static and extremely low-frequency magnetic fields, the flux shunt effect of high-permeability materials plays a dominant role. Lu et al. proposed a high-precision voltammetry method for the magnetic properties of permalloy under ultra-low-frequency magnetic field for multi-layer magnetic-shielding devices, which provides the necessary data support for the analysis of shielding performance at low frequency [51]. With the increase in the magnetic field frequency, the eddy current effect of the high-conductive materials becomes more and more obvious, and gradually replaces the role of high-permeability materials. It is worth noting that if the gap in the shielding layer is too large, resulting in poor conductivity, the shielding performance may show a downward trend with the increase in frequency, which is due to the weakening of eddy current effect caused by the gap and the obvious decrease in magnetic permeability with increasing frequency.

Silicon steel and permalloy are regular soft magnetic materials used for magnetic-shielding devices. Among all the soft magnetic materials, silicon steel is the most used material due to its low cost and its excellent electrical and magnetic properties. The widely used compositions for silicon steel are 6.5 wt.% Si and 3.2 wt.% Si, both of which possess excellent soft magnetic properties, with high-saturation magnetization of approximately 1.8 T and 2.0 T and high electrical resistivity of approximately 82 μΩ∙cm and 60 μΩ∙cm [52]. However, by reason of its relative low permeability (7000~10,000), when it comes to constructing a magnetic-shielding device with a specialized structure, such as the open-type structure, it is necessary to ensure the shielding performance for low-frequency fields [53]. Additionally, the shielding effect varies between grain-oriented and non-oriented silicon steels, which further increases complexities and difficulties in the process and construction sections [54]. Permalloy, also known as iron nickel alloy, mainly contains nickel (30~90 wt.%), as well as other additions like iron, manganese, silicon, molybdenum, and cobalt. 80Ni-permalloy grades possess prominent soft magnetic properties with magnetic permeability of a high level, coercivity adjustable in a wide range from 0.25 A/m to kA/m level, and pronounced cold-rolled ability for fabrication. With a content of 75–80 wt.% Ni, the initial relative permeability can even reach over 100,000 [20,55]. Accordingly, permalloy has become the most popular choice for magnetic-shielding devices [56,57,58]. 

Shielding factor (SF) is the crucial indicator to evaluate the magnetic shielding performance, which are defined as the ratio of the magnetic field strength or magnetic induction strength without shielding and after shielding at a certain position. The SF test often requires the construction of a uniform testing coil outside the magnetic shielding room to generate testing magnetic fields with different frequencies. Besides, it involves calculations based on coil constant to obtain the before-shielding magnetic field and measurements inside the magnetic-shielding devices to obtain the after-shielding magnetic field. The geometric shapes and structure dimensions of magnetic-shielding devices have great influences on the overall shielding effect. The theoretical calculation formulas for the static and low-frequency shielding factors of magnetic-shielding devices with spherical, cylindrical, and cubic forms are shown in Table 1, which ignores the eddy current effect.

It is worthing noting that the DC SF is much higher than the value at an extremely low frequency (SF @0.01 Hz) due to degaussing, especially for the device with less shielding layers. Degaussing can adjust the state of the soft magnetic materials and eliminate the extra residual magnetic field generated by stress and transportation, which can also be calculated by introducing higher permeability of an anhysteretic magnetization curve into the above formulas. The principle of degaussing will be debated in the latter part of this article. Magnetic noise represents the dynamic magnetic field fluctuation in magnetic-shielding devices after shielding most environmentally alternating magnetic fields. The origin of magnetic noise is typically thought to be complicated. Tang et al. [36] proposed that the composition of the magnetic noise within the magnetic shielded room is composed of residual environmental noise, material noise, and additional noise. The residual environmental noise is determined by the SFs of the device, and the material noise is mainly caused by hysteresis dissipation and eddy-current dissipation, while the additional noise is attributed to the instrumental noise floor of sensors. The aggregate power spectrum of the above three types of magnetic noise can be measured directly. In the magnetic-shielding device, the multiple layers can shield most of the noise from external environment, which makes the inherent magnetic noise of the innermost material noise become one of the research highlights to improve the shielding properties. The calculation methods of magnetic noise and reviews about materials with low magnetic noise will be introduced in detail in Section 3.

Since the world’s first magnetic-shielding devices designed by BobJ. Patton and John L. Fitch from Socony Mobil Oil company established in 1962, magnetic-shielding devices have been designed and studied over the past decades. Currently, magnetic-shielding devices with high performance and relatively low cost show great potential among a wide range of applications, which provide ultra-low magnetic environment for the investigations and experiments of fundamental physics phenomena, such as electric dipole moments of fundamental systems (EDMs), violation of Lorentz invariance and spin-couplings. Magnetic-shielding technologies also promote the research for medical magnetic-imaging techniques such as MCG and MEG. Here, we briefly list the most famous magnetic-shielding devices and their typical performances in Table 2.

## 3. Magnetic-Shielding Enhancement Through Low-Noise Materials

Magnetic noise of the nearly zero-field environment is a significant evaluation indicator for magnetic shields. For the muti-layers magnetic-shielding device with high performance, the magnetic noise from the shielding materials is the main noise source, which directly determines the sensitivity of magnetic measurement. Therefore, it is necessary to select low-noise soft magnetic materials as the innermost shielding layer to create the extremely weak magnetic environment. In this chapter, the calculation method of magnetic noise is deduced based on the fluctuation–dissipation theorem and Bertotti loss separation model, and some common low-noise materials are also introduced, including ferrites, amorphous, and nanocrystalline.

### 3.1. Principle and Methods of Material Noise Calculation

#### 3.1.1. Fluctuation–Dissipation Theorem

Assuming a circular-coil-applied excitation current is close to the conductive slab, the induced current of the conductive plate will cause a fluctuating voltage across the terminals of this coil. Magnetic noise can be understood as power loss incurred by the circular coil, and the spectral density of magnetic noise will be related to the impedance [68]. The whole process of induction can be described as follows: Firstly, the excitation current generates a *B*-field around the coil and its time rate of change induces a toroidal *E*-field. Subsequently, currents are induced in turn within the slab forming a return flux loop that connects the induced slab currents back to the coil. Moreover, the return flux induces a phase-lagged coil voltage equivalent to that of a resistive impedance, thus ensuring that the energy externally supplied to excite the coil balances the energy dissipated in the slab. Based on the fluctuation–dissipation theorem [69] which was developed from the generalized Nyquist relationship, the energy-spectral density of magnetic noise under the driving magnetic field can be calculated from power loss caused by the oscillating current.

Here, we briefly introduce the principle of fluctuation–dissipation theorem. According to Faraday’s law, a magnetic field fluctuation given by its power-spectral density of *S_B_*(*f*) will induce a voltage fluctuation on a coupling coil with a sufficiently small area at the same position, generating the power spectral density:

(1) $$S_{v}(f)=A^{2}N^{2}\omega^{2}S_{B}(f)$$

where *A* refers to the area of the *N*-turn pickup coil and ω=2πf. Ideally, the coil does not cause extra fluctuation and is completely conductive, which means the power loss is completely caused by the electromagnetic losses of materials. And therefore, the voltage fluctuation under the thermal equilibrium conditions can be described as follows:

(2) $$S_{v}(f)=4kTR_{eff}(f)$$

where *R_eff_*(*f*) is the real part of the impedance *Z*, *T* is the temperature in Kelvins, and *k* is the Boltzmann constant. The time-averaged power dissipation is obtained from the following:

(3) $$P(f)=\frac{1}{2}I^{2}R_{eff}(f)$$

where I(t)=Isin(ωt) represents the oscillating current flowing in the pickup coil. Combining Equations (1)–(3), the calculation formula of magnetic noise is described as follows:

(4) $$\delta B(f)=\sqrt{S_{B}(F)}=\frac{\sqrt{4kT}\sqrt{2P(f)}}{ANI\omega }$$

The fluctuation–dissipation theorem directly calculates the magnetic noise based on energy loss and avoids vector calculations of different magnetic noise sources inside the material, which effectively simplifies the computational complexity. For magnetic materials, the energy loss mainly originates from two sources, the eddy current loss caused by the thermal motion of electrons inside the conductors (also known as Johnson noise or Nyquist noise [70]) and the hysteresis loss caused by the movement and reorientation of magnetic domain walls [71] driven by an external magnetic field. The abnormal loss (also called excess loss) of magnetic-shielding materials at low frequencies can be ignored.

The eddy current loss at low frequency and the hysteresis loss can be expressed as follows:

(5) $$P_{eddy}=\int_{V}\frac{1}{2}\sigma E^{2}dV$$

(6) $$P_{hyst}=\int_{V}\frac{1}{2}\omega \mu^{\prime \prime }\sigma H^{2}dV$$

which, respectively, represent the volume integral of the electric and magnetic fields in the material introduced by the oscillating current *I*(*t*). Here, *σ* is the material’s conductivity, *E* is the electric field intensity, *H* is the magnetic field intensity, *μ* is the material’s magnetic permeability, and $\mu^{\prime \prime }$ is the imaginary part of the magnetic permeability caused by hysteresis loss [72]. And therefore, the calculation of energy dissipation involves two procedures. The first part is to calculate the volume integration of electromagnetic and magnetic fields in magnetic-shielding materials based on assumed excitation currents, which corresponds to the $\int_{V}E^{2}dV$ and $\int_{V}H_{m}^{2}dV$ in Equations (5) and (6). In simple geometries with symmetrical structures, analytical expressions for the magnetic noise can be obtained [31]. Analytical formulas for the common symmetric geometries are summarized in Table 3. In complicated geometries or for traverse calculations, semi-analytical or numerical methods [73] are employed to compute the magnetic noise with the help of a finite element model (FEM) software [74,75,76]. And the second part of calculation involves the value of $\mu^{\prime \prime }$. The complex magnetic permeability obtained under alternating magnetic fields is usually the comprehensive result of multiple losses. And therefore, loss separation is necessary to obtain the imaginary part of permeability caused by hysteresis loss, which corresponds to $\mu^{\prime \prime }$ in the Formula (6). Consequently, we review the research in magnetic permeability testing based on loss separation.

#### 3.1.2. Loss Separation

**Figure 2:**
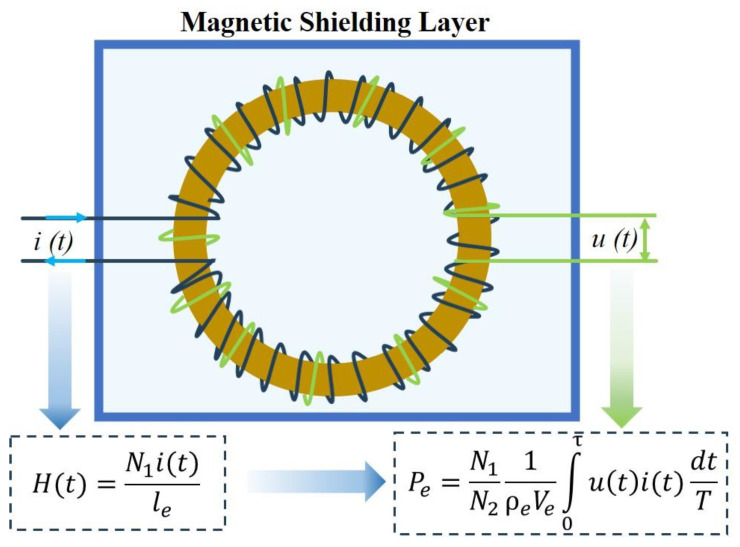
Schematic of the permeability measurement using voltammetry.

Impedance analysis is a widely used method to measure the material’s complex permeability by relating the real part and imaginary part with the inductance and resistance of the equivalent sample series circuit, respectively, as follows [78]:

(7) $$\mu^{\prime }=\frac{l_{e}}{\mu_{0}A_{e}N^{2}}L_{s}$$

(8) $$\mu^{\prime \prime }=\frac{l_{e}}{\mu_{0}A_{e}N^{2}\omega }R_{s}$$

where *L_s_* and *R_s_* are relatively the inductance and resistance of the equivalent series circuit, *A_e_* is the equivalent cross-section area of the testing sample, *N* is the number of turns of the coils, *l_e_* is the equivalent length of the magnetic path, *ω* is the angular frequency of the driving field, *μ_0_* is the permeability of vacuum, and $\mu^{\prime }$ and $\mu^{\prime \prime }$ are the real part and imaginary part of the relative complex permeability, respectively. For materials with extremely low conductivity, such as ferrites, eddy current loss is negligible, and the measured imaginary part of magnetic permeability is considered to entirely come from hysteresis loss. However, for magnetic materials that cannot ignore eddy current loss, such as permalloy, loss separation based on voltammetry is necessary to obtain the $\mu^{\prime \prime }$ to solve Equation (6). The measurement principle is shown in Figure 2.

The loss characteristics of ferromagnetic materials can be described and solved through mathematical models and empirical analytical formulas. The commonly used mathematical models are Preisach model, Jiles–Atherton (J–A model), and their derivative models. The Preisach model [79] assumes that ferromagnetic materials are composed of plenty of magnetic domain units with rectangular hysteresis characteristics, and the hysteresis loop can be described as follows:

(9) $$M(t)=\iint_{H_{u}\geq H_{d}}\phi \left(H_{u},H_{d}\right)*\hat{m}(H_{u},H_{d})*H(t)dH_{u}dH_{d}$$

where the Preisach distribution function $\varphi \left(H_{u},H_{d}\right)$ characterizes the density of magnetic domains with *H_u_* and *H_d_* as the positive and negative magnetic field thresholds of the hysteresis loop, respectively, and $\hat{m}=\left(H_{u},H_{d}\right)$ is hysteresis factor with a value of +*m_s_* or −*m_s_*. The Preisach model requires a large amount of measurement data for adapting and fitting, and the form of double integration increases the computational difficulty. The J–A model predicts the magnetization process as the movement of magnetic domains and calculates the energy balance equation based on the displacement and substitution of magnetic domain walls. The J–A model [80] comprehensively considers irreversible changes such as pinning effects and reversible changes such as elastic deformation during the movement of magnetic domain walls to reveal the magnetization characteristics of materials based on physical phenomena. There are five parameters involving in the differential equations of the J–A model, with *Ms* as the saturation magnetization, *α* as the interdomain coupling coefficient, *a* as the shape parameter for anhysteretic magnetization, *c* as the domain wall flexibility coefficient, and *k* as the pinning coefficient [81,82,83]. The static magnetic model equations are transformed as the following expression [84]:

(10) $$\frac{dM}{dB}=\frac{\delta (M_{an}-M)+ck\delta \frac{dM_{an}}{dH_{e}}}{\mu_{0}\left[k\delta +(1-\alpha )\left(\delta (M_{an}-M)+ck\delta \frac{dM_{an}}{dH_{e}}\right)\right]}$$

(11) $$\frac{dM_{an}}{dH}=\frac{M_{s}}{a}\left[1-\coth^{2}\left(\frac{H_{e}}{a}\right)+{\left(\frac{a}{H_{e}}\right)}^{2}\right]$$

where *M* and *M_an_* represent the total magnetization and anhysteretic magnetization, *H_e_* = *H* + *αM* represents the effective field, and *δ* is the direction parameter used to ensure that the pinning opposes change in magnetization. Based on the dissipation theory of Bertotti, the J–A model under dynamic magnetic fields takes into account the eddy current and is modified as follows [85]:

(12) $$\begin{array}{c} \left(\frac{\mu_{0}d^{2}}{2\rho \beta }\frac{dM}{dt}\right){\left(\frac{dM}{dH}\right)}^{2}+{\left(\frac{Gdw\mu_{0}H_{0}}{\rho }\right)}^{1/2}{\left(\frac{dH}{dt}\right)}^{1/2}{\left(\frac{dM}{dH}\right)}^{3/2}\\ +\left[k\delta -\alpha \left(M_{an}(H)-M(H)+k\delta c\frac{dM_{an}}{dH_{e}}\right)\right]\left(\frac{dM}{dH}\right)\\ -\left(M_{an}(H)-M(H)+k\delta c\frac{dM_{an}}{dH_{e}}\right)=0 \end{array}$$

where *ρ* is the resistivity in Ω·m, *d* is the cross-sectional dimension in meters, and *β* is a geometrical factor. These five J–A parameters are usually obtained by fitting measurement data of saturated *B*-*H* loop with the utilization of optimization algorithms, including particle swarm optimization [86], genetic algorithm [87], shuffled frog-leaping algorithm [88], and simulated-annealing algorithm [89]. Xu et al. [90] proposed the variable-temperature dynamic Jiles–Atherton model to compute and separate the loss of nanocrystalline 1K107 sample rings. Losses under various temperatures were successfully separated.

The empirical analysis formulas mainly include Steinmetz empirical formula, G. Bertotti loss separation model, and their developed calculation models. Steinmetz et al. [91] first summarized the generic computation for hysteresis and eddy-current losses, which was simplified as the Steinmetz equation or original Steinmetz equation (OSE) [79]:

(13) $$P_{OSE}=kf^{\alpha }Bs^{\beta }$$

where *P_OSE_* is the time-average core loss per unit volume, *k* is a material parameter, *f* is the frequency, *Bs* is the peak induction of the sinusoidal excitation, and *α* and *β* are the frequency and magnetic induction exponents, respectively. After decades of development, various models based on this theory have been derived to achieve loss calculation under complex excitations, such as Modified Steinmetz Equation (MSE) [71], General Steinmetz Equation (GSE) [92], Doubly Improved Steinmetz Equation [93], etc. The Bertotti loss separation model defined the core loss as the sum of the hysteresis loss, the eddy current loss, and the anomalous or excess loss, as given by the following [94]:

(14) $$\begin{array}{c} P_{total}=P_{hyst}(f)+P_{eddy}(f)+P_{ex}(f)\\ =k_{h}fB_{m}^{\alpha }+k_{e}f^{2}B_{m}^{2}+k_{ex}f^{1.5}B_{m}^{1.5} \end{array}$$

where *k_h_*, *k_e_*, and *k_ex_* are coefficients of hysteresis loss, eddy current loss, and excess loss respectively, *α* is Steinmetz coefficient, and *B_m_* is the peak value of the flux density amplitude. The eddy current loss and excess loss are defined as follows [79]:

(15) $$\begin{array}{l} Peddy=\frac{\pi^{2}d^{2}f^{2}B_{s}^{2}}{\rho \beta }\\ P_{ex}=8\sqrt{\frac{GAV_{0}}{\rho }}B_{s}^{1.5}\sqrt{f} \end{array}$$

where *d* is the lamination thickness, *ρ* is the electrical resistivity, *β* is the magnetic induction exponent, *G* is about 0.2, *A* is the cross area of the lamination, and *V*_0_ is a parameter that characterizes the statistical distribution of the magnetic objects responsible for the anomalous eddy currents. Ma et al. [25] used the method of the Bertotti’s loss separation to prove that the hysteresis loss is the dominant magnetic noise sources for ferrite shield below 100 Hz. Xu et al. [72] accurately calculated the imaginary-part permeability caused by hysteresis losses using the Bertotti model considering the influence of eddy currents.

### 3.2. Materials with Low Noise

#### 3.2.1. Spinel Ferrites

**Figure 3:**
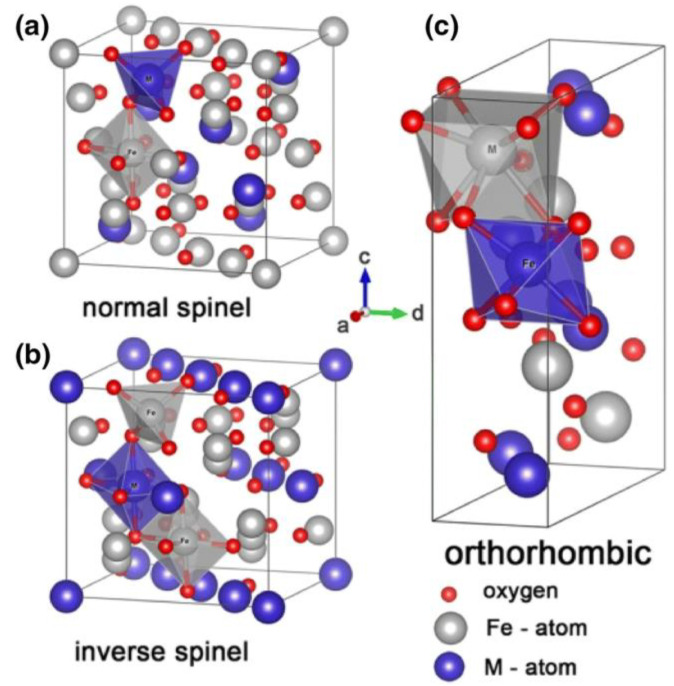
Crystal structures of spinel ferrites: (**a**) normal spinel, (**b**) inverse spinel, and (**c**) orthorhombic, each demonstrating the three crystallographic sites [105].

Ferrites [95,96] are a large class of ceramic materials composed of iron oxide (Fe_2_O_4_) and metallic elements like Ba, Mn, Ni, Zn, etc., in specific proportions. Based on the crystal structure, ferrites can be classified into spinel, garnet, ortho, and hexagonal ferrites [97]. The general formula for spinel ferrites is MFe_2_O_4_ [98] or MO∙Fe_2_O_3_ [99], where M represents individual metal ions, such as Mn, Ni, Zn, Mg, Co, Cu, or a combination of cations, such as Mn-Zn, Ni-Zn, etc. Due to their high permeability and high electrical resistivity, spinel ferrites have been variously used in electromagnetic interference shielding and soft magnetic devices [100]. The unit cell of spinel ferrite consists of a face-centered cubic (FCC) containing eight sub-cells, with two types of vacancies: 64 tetrahedral sites (A sites) surrounded by four oxygen ions and 32 octahedral sites (B sites) surrounded by eight cations [101,102]. Only 8 tetrahedral sites and 16 octahedral are occupied by the metal sites cations ultimately [103,104]. Depending on the distributions of M and Fe ions between tetrahedral (A) and tetrahedral (B) sites, spinel ferrites can be categorized into normal spinel, inverse spinel, and mixed spinel; their crystal structures are shown in Figure 3 [105].

The magnetic properties of ferrites are influenced by the unpaired 3d electrons of cations, super exchange interactions between adjacent metal ions, and the occupancy distribution of different sites [103,106,107,108]. Kumar et al. [98] summarized the effects of crystal structure, grain size, porosity, additives, and various crystal defects on the initial magnetic permeability of ferrites. Thakur et al. [101] reviewed the research progresses on the magnetic properties of Mn-Zn ferrites, including saturation magnetization, remanence, and coercivity. Ferrites can be divided into soft magnetic materials with low coercivity and hard magnetic materials with high coercivity. Soft magnetic materials, characterized by high magnetic permeability, high resistivity, and low losses, are commonly used in the electronics industry. The most common soft magnetic ferrite materials are Mn-Zn ferrites (Mn_1-x_Zn_x_Fe_2_O_4_) and Ni-Zn ferrites (Ni_1-x_Zn_x_Fe_2_O_4_), with Mn-Zn ferrites having a relative permeability of 800–15,000 and electrical conductivity 5–7 times smaller than that of μ-metal. The relative permeability of Ni-Zn ferrites ranges from 10 to 2000. The magnetic properties of ferrites are controlled by comprehensive combinations of mechanisms including magnetocrystalline anisotropy, surface anisotropy, and inter-particle interactions. Therefore, various synthesis methods with different preparation conditions, including synthesis temperature, cation concentration, and particle size, make significant differences in the magnetic properties of Mn-Zn ferrites [109,110,111]. Table 4 summarizes the magnetic properties of Mn-Zn ferrites.

Up to now, there have been numerous reports of sensitive measurements based on the magnetic environment with high accuracy provided by ferrites as part of the magnetic-shielding devices. Mn-Zn ferrites with various oxide additives become the most common ferrite materials for magnetic-shielding devices. By applying a cylindrical magnetic shield composed of three layers of μ-metal surrounding a ferrite shell, an ultra-low magnetic noise of 0.75 fT/Hz^1/2^ was in situ measured by a SERF atomic magnetometer, which was 25 times lower than a complete μ-metal shield in the same size [77]. Fan et al. proved the great advantages of ferrites as the inner shield of the K-Rb-^21^Ne co-magnetometer by comparing the temperature sensitivity of different shielding materials [120]. Pang et al. proposed a temperature suppression method to reduce the thermal magnetic noise of the ferrite. The magnetic noise at 1 Hz was successfully decreased from 15.24 fT/Hz^1/2^ to 8.12 fT/Hz^1/2^, which significantly improved the sensitivity of the co-magnetometer from 1.21 × 10^−5^ °/s/Hz^1/2^ to 7.02 × 10^−6^ °/s/Hz^1/2^ [121]. Through optimizations of coils structure and degaussing parameters, the cubic ferrite shield performed an averaged magnetic noise of 2.62 fT/Hz^1/2^ at the center point [122].

#### 3.2.2. Iron-Based Amorphous and Nanocrystalline

Amorphous alloys are fabricated through rapid cooling below the glass transition temperature of metallic melts. Owing to the short-range ordered but long-range disordered atomic structure and the obvious dense regions of nearest-neighbor and second-nearest-neighbor atoms, amorphous alloys exhibit completely different performance compared with crystal materials. Despite the disordered packing geometries in long range, some short-range-order structures connect with each other to form crystal-like superclusters and give rise to medium-range (0.5~2 nm) orders [123]. Cheng et al. [124] provided a detailed overview of the structure characteristics of metallic glasses and the common structure models of an amorphous solid like microcrystalline model, continuous random network model, dense random packing of hard spheres [125], and dense cluster packing [126] model. The microstructural characteristics of amorphous alloys enable better soft magnetic performances. Crystal defects, including grain boundaries, phase boundaries, and dislocations, do not exist within amorphous alloys. And the magnetic domains can easily move, reorientate, and expand without being affected by the pinning effect, thus exhibiting lower coercivity. The long-range disordered structure causes a random distribution of internal magnetic moments with approximately equal atomic density in all directions. The magnetization ability is similar in each direction which greatly reduces the magneto-crystalline anisotropy constant. However, the Achilles’ heel of amorphous alloys is its weak ductility, with a room-temperature plastic deformation less than 2%, which severely restricts its further expansion and application [127,128,129].

Amorphous and nanocrystalline alloys can be formed by precipitating nano-sized particles in the amorphous matrix after certain special heat treatment. By controlling uniform distributions of ferromagnetic nanoparticles embedded on the amorphous matrix, the dual phase structure of both amorphous and nanocrystal alloys can achieve higher saturation magnetization and higher effective magnetic permeability compared with the single amorphous phase. The magnetic properties of amorphous and nanocrystalline alloys are related to magneto-crystalline anisotropy energy [130]. Amorphous materials have almost no magneto-crystalline anisotropy energy, while the magneto-crystalline anisotropy energy of nanocrystalline materials is much lower than traditional soft magnetic materials [128]. Meanwhile, the unique laminated structure effectively suppresses eddy current losses, making it one of the best choices for magnetic shielding.

The random anisotropy model proposed by G. Herzer et al. [130,131] indicates that the magnetic properties of amorphous and nanocrystalline alloys depend strongly on the counterplay of local magnetic anisotropy energy and ferromagnetic exchange energy. For large grains, the magnetization process mainly relies on the magneto-crystalline anisotropy *K*_1_ of the crystallites. The magnetization intensity follows the easiest magnetized direction in the single grains, and domains can be formed inside the grains. As for very small grains, the ferromagnetic interaction can be strengthened and strongly forces the magnetic moments to align parallel, thus preventing magnetization density from following the easy magnetization axis within a single grain. In another word, the effective magnetic anisotropy is the averaged value among several grains. The effective ferromagnetic exchange length can be expressed as follows:

(16) $$L_{ex}^{0}=\sqrt{\frac{A}{K_{1}}}$$

where *A* represents the exchange stiffness, *K*_1_ represents the magneto-crystalline anisotropy constant, and $L_{ex}^{0}$ represents the effective ferromagnetic exchange length over which the magnetization will vary appreciably. And the coercivity can be expressed as follows:

(17) $$H_{e}=P_{c}\frac{\langle K\rangle }{J_{s}}\approx P_{c}\frac{K_{1}^{4}D^{6}}{J_{s}A^{3}}$$

And permeability can be expressed as follows:

(18) $$\mu_{e}=P_{\mu }\frac{J_{s}^{2}}{\mu_{0}\langle K\rangle }\approx P_{\mu }\frac{J_{s}^{2}A^{3}}{K_{1}^{4}D^{6}}$$

where $\langle K\rangle$ denotes the effective magneto-crystalline anisotropy constant, which represents the averaged anisotropy amplitude among the grains within the effective exchange range, $J_{s}$ denotes the magnetization intensity, $P_{c}$ and $P_{\mu }$, respectively, denote the displacement of magnetic domain walls at 180° and 90°, *D* denotes the grain size, and *A* denotes the exchange stiffness. When the grain size is smaller than ferromagnetic exchange length, the coercivity decreases with the grain size, while the permeability responds oppositely.

**Figure 4:**
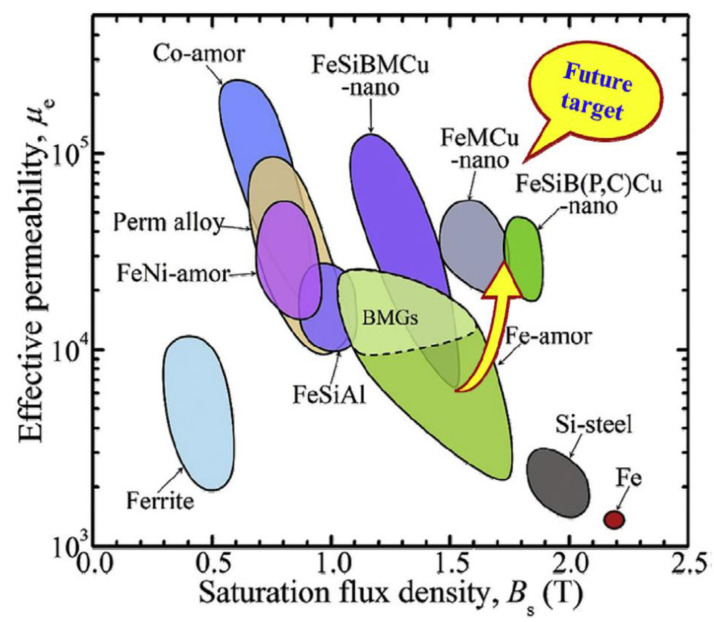
Relationship between effective permeability (*μ_e_*) at 1 kHz and saturation flux density (*B_s_*) for soft magnetic materials [127].

Among the amorphous matrix of ferromagnetic elements, iron-based alloys have higher saturation magnetic density, higher permeability, and lower coercivity, making them ideal soft magnetic materials [127]. Depending on the crystal structure of nanoparticles, amorphous and nanocrystalline alloys can be classified into four categories [132]: (1) α-Fe_3_Si-based alloy featuring a DO_3_ crystal structure, typically composed of Fe-Si-B-M-Cu (M = Nb, Mo, W, Ta, etc.), with the grade as Finemet. (2) α-Fe-based alloy featuring a BCC (A2) crystal structure, typically composed of Fe-M-B-(Cu) (M = Zr, Hf, Ta), with the grade as Nanoperm. (3) α’-Fe_50_Co_50_-based alloy featuring a B2 crystal structure, typically composed of (Fe,Co)-M-B-Cu(M = Zr, Hf, Nb, etc.), with the grade as Hitperm. (4) New bcc-Fe (Si)-based alloy typically composed of Fe-Si-B-P-Cu, with the grade of Nanomet. The relationship between the effective magnetic permeability at 1 kHz and saturation magnetization of various alloys is depicted in Figure 4 [127]. 

Finemet alloy was first found by Yoshizawa [133] in 1988. Fe_73.5_Si_13.5_B_9_Cu_1_Nb_3_ was prepared through the addition of Cu, Nb, and other metal elements into the Fe-Si-B amorphous alloy system. After annealing crystallization, randomly orientated α-Fe (Si) nanoparticles with grain size of 5–15 nm were formed. The saturation magnetization reached 1.24 T, coercivity and core loss were greatly decreased, while permeability was significantly improved. Currently, Finemet alloys are widely applied in commercialization. After that, Suzuki [134] began to study and prepare Nanoperm alloys. The permeability of synthesized series alloys exceeds 10,000 at 1 kHz, and the saturation magnetization is as high as 1.5 T. Furthermore, the core loss of Fe_86_Zr_7_B_6_Cu_1_ was decreased to 0.066 W/kg under the condition of 1 T and 50 Hz. Conde et al. [135] adjusted the element composition of FeMoBCu series alloys (Nanoperm type) to control the thermal stability and concluded the variation regulation of room-temperature saturation magnetization with the composition of alloys. R. Parsons et al. [136] achieved the excellent soft magnetic performances of coercivity values low as 1.5~6 A/m and saturation magnetic polarization values high as 1.64–1.92 T by utilizing a rapid annealing process upon the Fe-Nb-B-(Cu) series Nanoperm alloys with a Nb content less than 4 at.%. Małlkiński et al. [137] found the crystalline phase fractions of Fe_89_Zr_7_B_4_ can be related to heat treatment temperature, and the shape of hysteresis loop, coercivity, and magnetic permeability were bound up with the proportion of nanocrystals in the alloy. A fraction of crystalline over 60% can lead to better soft magnetic properties. Willard et al. [138] prepared an (Fe_0.5_Co_0.5_)_88_Zr_7_B_4_Cu_1_ Hitperm alloy with higher saturation magnetization than the Nanoperm alloy. Škorvánek et al. [139] also investigated the impacts of field-annealing on nanocrystalline Hitperm alloys. By adding P element, the grain size of α-Fe for Fe_85_Si_2_B_8_P_4_Cu_1_ [131]. The Nanomet alloy was decreased to 20 nm, with saturation magnetization and coercivity values reaching 1.85 T and 5.8 A/m. The investigation on the nucleation mechanism of nanocrystallinity [140] in Nanomet alloys indicated that the grain size was determined by the growth competition between present and newly generated nuclei, and a model of nano crystallization was proposed to tailor the nanostructure for Nanomets. Table 5 reviews the compositions and magnetic properties of amorphous and nano-crystalline alloys mentioned above.

Iron-based amorphous and nanocrystalline alloys are new promising shielding materials for magneto-sensitive measurements. Fe-Nb-Cu-Si-B nanocrystalline alloys have been widely applied in magnetic-shielding improvements. Fu et al. first applied nanocrystalline as the shielding part in a co-magnetometer and verified its feasibility [3]. Liu et al. compared the magnetic noise generated by amorphous and nanocrystalline shields with varying layers. Nanocrystalline was tested as the best choice for SERF co-magnetometers due to its lower thermal magnetization noise, better temperature stability and laminated structure [145]. By establishing a theoretical magnetic noise model of nanocrystalline shields, the structural parameters optimization for low-noise shields was supported to realize better the sensitivity of co-magnetometers [146]. Xu et al. simulated the magnetic noise of 1K107 nanocrystalline in the SERF magnetometer and derived the imaginary parts of permeability [90].

## 4. Magnetization State Control for Shielding-Performance Improvement

The performance of a magnetic-shielding device is mainly determined by the magnetic properties of high permeability soft magnetic material, especially in the low-frequency band. Due to the hysteresis effect, the magnetic characteristics of magnetic-shielding materials are often nonlinear and directly affected by external conditions of magnetic excitation and environment [147]. Therefore, the magnetization states of the shielding materials can be controlled by the electromagnetic excitation methods, such as degaussing and magnetic shaking, which can be used to effectively enhance the magnetic properties of the material and the overall shielding performance of the device. In this chapter, the principles and calculation methods of degaussing and magnetic shaking are introduced, and the practical applications in magnetic-shielding devices are listed in detail.

### 4.1. Degaussing and Magnetic Equilibration

#### 4.1.1. Magnetic Equilibration Principle of Degaussing

Materials with high magnetic permeability in magnetic-shielding devices are highly susceptible to magnetization during transportation and installation. Due to the hysteresis characteristics, magnetic domains retain their original orientation even after the external magnetic field and stress are removed, rather than returning to a random orientation, which results in additional residual magnetism in the magnetic-shielding layers. The additional magnetization of the material increases the residual field within the device, thereby reducing its effectiveness in shielding against external magnetic fields. Degaussing technology enables shielding materials to operate in an optimal magnetization state, making it a crucial step in enhancing the magnetic-shielding performance [39,148].

Common degaussing methods can be categorized into thermal and electromagnetic methods [149]. Thermal degaussing involves heating the magnetic material above its Curie point and then cooling it in the zero-field environment, effectively disrupting the magnetic domain direction and eliminating additional magnetization [150]. However, this method can reduce the precision of zero-magnetic device components and compromise structural integrity. Additionally, it is impractical for large-scale devices due to the limitations in the size of annealing furnace. Electromagnetic degaussing methods are further divided into direct current (DC) degaussing and alternating-current (AC) degaussing. The AC degaussing method is commonly used method in practical applications, such as vessels [151,152], magnetic recording [153], electron component, and magnetic shields [122,154]. It involves applying an alternating magnetic field which continuously changes the orientation of the magnetic domains within the magnetic material. By gradually reducing the amplitude of the degaussing field or moving the workpiece away from the alternating field, the residual magnetization in the magnetic materials is progressively weakened. 

**Figure 5:**
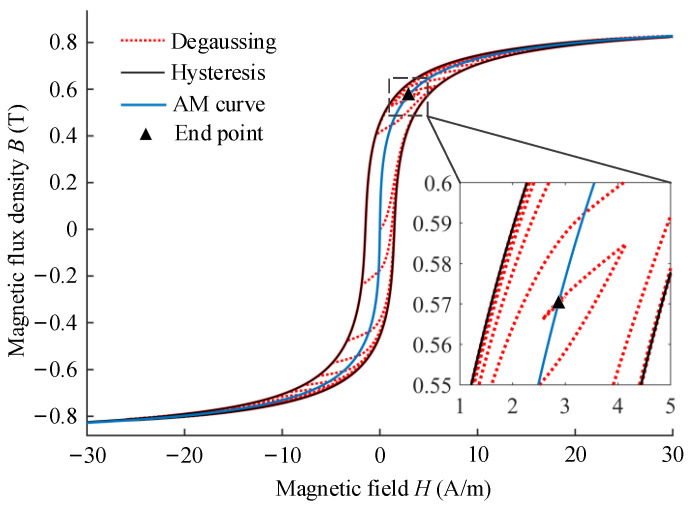
Magnetization state in the process of degaussing [147].

For the soft magnetic materials with high permeability of magnetic-shielding devices, the principle of degaussing is different from the traditional definition, the elimination of remanence inside magnetic material. The essence of flux shunt principle of magnetic shielding is that the high permeability material is magnetized by the external environmental field which is adsorbed in the shielding layers to avoid introducing into the device. Therefore, the residual magnetization of the material cannot be eliminated by degaussing in geomagnetic environment. In 2014, Altarev et al. proposed the principle of magnetic equilibration to describe the degaussing process [26]. The degaussing of magnetic shields cannot remove the remanence as the name suggests but rather adjusts the magnetic field in the material with respect to the external condition. The shielding performance for static magnetic field can be effectively improved through re-establishing the equilibrium between the magnetization of magnetic-shielding material and the static component of environmental field. Under the effect of degaussing field, the magnetic domain walls oscillate repeatedly, overcome the loss of the pinning effect constantly, and finally be closed to the state with the lowest potential energy, the anhysteretic state, as shown in Figure 5. The anhysteretic magnetization curve has higher initial permeability than the basic and initial magnetization curves, which also explains the inconsistency between the DC SF and the quasi-static SF of magnetic shield.

In 2016, Sun et al. described the magnetic equilibration procedure of degaussing based on the dynamic J–A model [155]. On the basis of previous studies, the anhysteretic curve model (AHCM), proposed by Sun et al. in 2021, is suggested as a replacement for the complex dynamic Jiles–Atherton model (DJAM) for the calculation of the static residual field inside magnetic-shielding devices [40]. However, the actual degaussing process cannot be as ideal as the AHCM calculation, and the residual field of MSR is often higher than the theoretical value due to the air gaps between the sheets of shielding layers. Note here that, the AHCM can only be used to estimate the DC shielding factor after degaussing, while the quasi-static shielding factor, such as 0.01 Hz, needs to be calculated by the initial magnetization curve model (IMCM). The anhysteretic magnetization curve can be tested by degaussing and re-magnetization under different bias DC fields, and the saturation magnetization is utilized as the calibration [156].

#### 4.1.2. Design of Degaussing Coil

**Figure 6:**
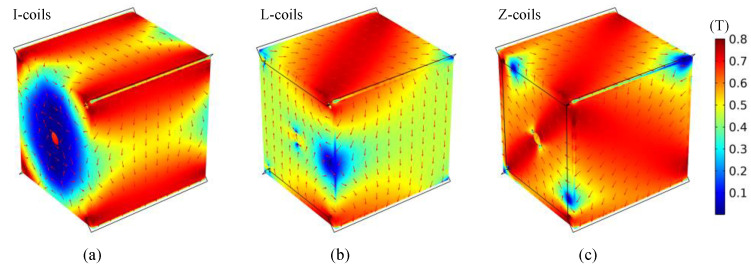
Simulation results of degaussing-field distribution of (**a**) I-coils, (**b**) L-coils, and (**c**) Z-coils.

The degaussing coil needs to ensure that the generated field is evenly distributed in the shielding layer as much as possible, which directly affects the static shielding performance of the magnetic-shielding device after degaussing. For the MSR, it can be simply divided into edge-type and distributed-type coils according to the position where the degaussing coil wound on the shielding layer. Through winding, the coil on the four parallel edges of MSR, the degaussing field can be introduced into the four corresponding walls of the shielding layer. By this method, a complete annular magnetic circuit is established, which is called the “I-coils” [39]. Using the same method, winding the coil on eight or twelve edges can realize the degaussing of all the planes of the shielding layer. These two methods are called “L-coils” [26] and “Z-coils” [154], which can be regarded as the combination of “I-coils” in different directions. The simulation of degaussing field distribution of these coils is shown in Figure 6. Obviously, the degaussing field generated by the I-coils is only distributed on the magnetic circuit composed of four wall surfaces as shown in Figure 6a. For the L-coils and Z-coils, the degaussing field can be distributed on all the shielding layers of MSR, but there is still an obvious uneven distribution at the edges and corners, as illustrated in Figure 6b,c. Sun et al. proposed to use a unified hole to replace the holes in the three adjacent faces at the corner to wind the degaussing coil, which can effectively avoid the magnetic field accumulation at the corner [157]. In order to compensate for the degradation of shielding performance caused by the large holes, the edges can be beveled as chamfers to improve the shielding effect.

**Figure 7:**
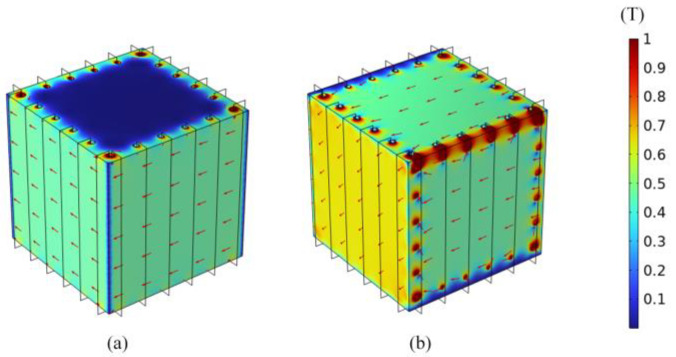
Simulation of degaussing field of (**a**) distributed I-coils and (**b**) distributed L-coils.

**Figure 8:**
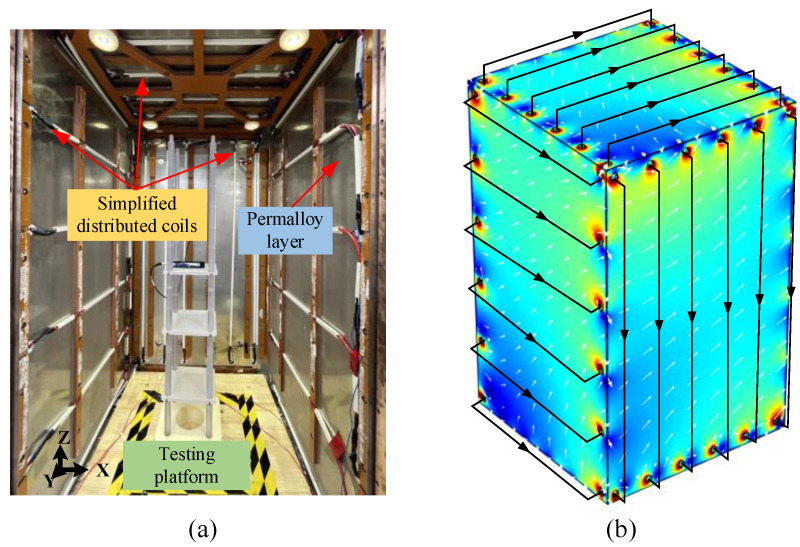
Simplified distributed-type coils of the magnetic shielding booth: (**a**) photo of the interior; (**b**) simulation of degaussing field [158].

During magnetic equilibration, the oversaturated areas at the edges and corners come out of saturation later than other areas, leading to an insufficient number of effective degaussing cycles. In order to improve the uniformity of degaussing field and avoid high flux density at edges and corners of layer, Sun et al. proposed the distributed-type coils based on the edge-type coils in 2021 [40], as shown in Figure 7. Compared with the traditional degaussing coils in Figure 6, the degaussing fields generated by the distributed coils is more uniform, especially at the edges and corners. By this means of distributed-type coils, an unprecedented residual field of <130 pT was repeatedly achieved over 0.5 × 0.5 × 0.5 m^3^ inside a three-layer MSR. In 2023, Yang et al. adopted a simplified distributed-type coils for the magnetic shielding booth composed of two permalloy layers and one aluminum layer, as shown in Figure 8 [158]. The simplified distributed coil is wound only on two opposite wall surfaces to generate a toroidal degaussing field. In this way, three groups of the coils are wound to form complete magnetic circuit in the shielding layers. After degaussing, the residual field of 5.1 nT was achieved in the center of this shielding device. Due to the magnetic resistance caused by the door crack, the shielding door of MSR is difficult to form a complete magnetic circuit as other shielding layers. Ayres et al. wound the additional degaussing coils on the shielding door to ensure the integrity of the magnetic circuit [159].

**Figure 9:**
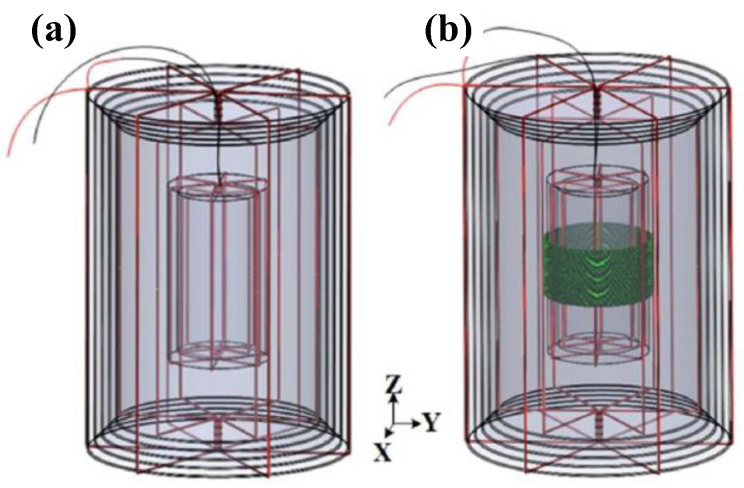
Configuration of the combined degaussing coils: (**a**) toroidal coil and (**b**) solenoidal coil [160].

Some low-noise magnetic-shielding materials, such as Mn-Zn ferrite, tend to have greater coercivity, resulting in difficulty in establishing magnetic equilibrium. Yang et al. wound the additional solenoidal coil on the ferrite layer based on the traditional toroidal coil, as shown in Figure 9, to reduce the residual field inside a cylindrical magnetic shield, which is composed of four outer permalloy layers and one inner Mn-Zn ferrite layer [160]. This degaussing coil composed of the toroidal coil and solenoidal coil ensures that each part of the shielding material reached the saturation magnetization almost simultaneously. By this method, a residual field below 0.6 nT and a gradient lower than 0.5 nT/cm were achieved along all three axes with ±20 mm at center.

#### 4.1.3. Optimization of Degaussing Parameter

The parameters of the degaussing-current waveform are also the crucial factors affecting the internal residual field after magnetic equilibration. The waveform of degaussing current often adopts sinusoidal signal, and its envelope function adopts linear attenuation function [39]. It is usually required that the initial amplitude of the degaussing current should make the magnetic-shielding material reach magnetic saturation to fully overcome the coercive force of the magnetic material, and the degaussing frequency should be reduced as far as possible to limit the unnecessary eddy current caused by the AC field. Increasing the number of degaussing cycles can effectively improve the effect of magnetic equilibration. However, when the number of cycles is large enough, the amplitude of degaussing current in the last few cycles may be submerged by current noise, and increasing the number of cycles is no longer effective at this time. It is worth noting that high-power power supplies of degaussing often have some DC bias, which greatly degrades the degaussing effect, so it is necessary to use isolated transformers to eliminate DC bias [161].

**Figure 10:**
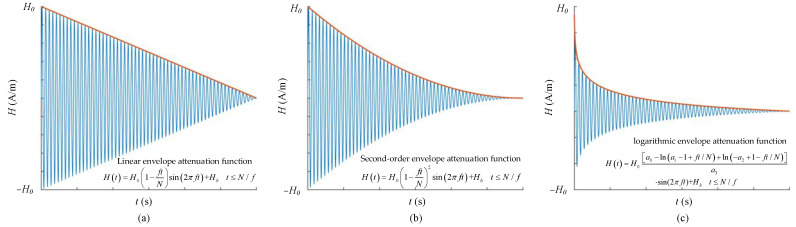
Degaussing waveform with different envelope attenuation functions: (**a**) linear attenuation, (**b**) second-order attenuation, and (**c**) logarithmic attenuation.

Due to the hysteresis effect of ferromagnetic materials, there are phase lags and some differences between the degaussing field and magnetic flux density inside the layer, which cannot be reduced linearly as the degaussing field, reducing the effect of magnetic equilibration. Therefore, it is necessary to establish the hysteresis model and characterize the degaussing process of different envelope attenuation functions through theoretical calculation, so that the magnetization in the material changes uniformly and linearly during the demagnetization process. Using a simplified hysteresis loop mathematical model, Thiel et al. simulated the variation of the magnetic flux density inside a material under the demagnetization field of a linear envelope decay function and proposed a new logarithmic envelope attenuation function to satisfy the requirement of short duration in the reversible interval of magnetic materials and long duration in the irreversible interval [162]. Sun et al. established the degaussing model of a single-layer shield based on the Jiles–Atherton model and optimized the envelope attenuation function of the degaussing current [155]. Using the exponential attenuation function instead of the linear function, the magnetic flux density in the material was reduced uniformly during the degaussing process, which reduces the residual field in magnetic shield. At present, the envelope attenuation function commonly used for demagnetization is shown in Figure 10, mainly including linear, second-order, and logarithmic functions. The blue lines represent the degaussing waveforms, and the red lines refer to the envelope decay functions. Compared with the linear functions, the other two envelope attenuation functions can achieve uniform reduction in the magnetic flux density in the magnetic-shielding material by first decaying rapidly and then slowly. Shi et al. designed a feedback control system for the degaussing-power supply to regulate the magnetic flux density inside the permalloy sheet as the sinusoidal wave with the linear envelope attenuation based on a single sheet tester (SST) [163]. By this way, the desired magnetization state of the shielding material can be controlled during degaussing. Later, Shi et al. also tested the nonlinear magnetic characteristics of the permalloy sheet, including basic magnetization and anhysteretic magnetization curves, for estimation of the residual field inside MSR [164].

**Figure 11:**
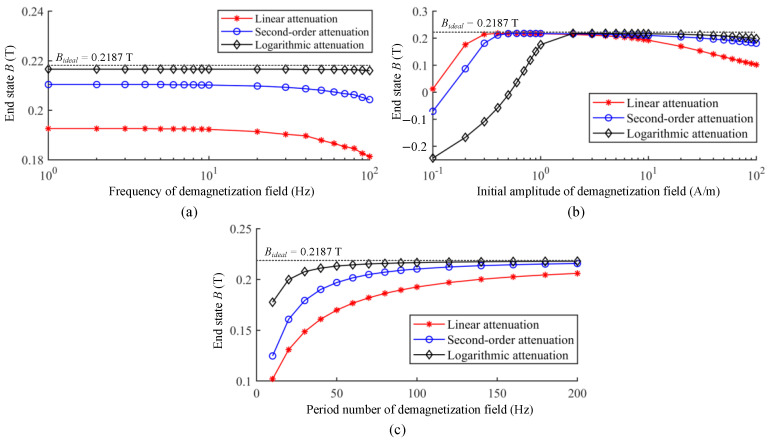
The end magnetization state of degaussing with different (**a**) frequencies, (**b**) initial amplitude, and (**c**) period number [158].

In the previous studies on degaussing, the static residual field inside magnetic-shielding device is always selected as the criterion to evaluate the effect of magnetic equilibration. However, the residual field of magnetic shield is also affected by the structure factors, which cannot directly reflect the mechanism of degaussing. Yang et al. proposed a new assessment criterion to analyze and evaluate the parameters of degaussing field based on the difference between the final magnetization after equilibration and the theoretical anhysteretic magnetization of the shielding material [158]. Based on the dynamic J–A model, the end magnetization states of degaussing with different parameters and envelope attenuation functions were calculated as shown in Figure 11. It is demonstrated that the low frequency, appropriate amplitude, sufficient period number, and logarithmic envelope attenuation function of the degaussing field facilitate the final magnetization state close to the corresponding anhysteretic state.

### 4.2. Magnetic Shaking

The method of magnetic shaking is similar to the AC electromagnetic degaussing, using a coil to apply an excitation magnetic field to the shielding layers. Therefore, these two methods can share the same set of coils. Their main difference is that an AC field with constant amplitude is introduced into the magnetically shielded layers to reduce the hysteresis loss of the shielding material and improve its initial permeability. Originally, this method was utilized for magnetic recording to overcome the nonlinearity of the recording medium caused by hysteresis [165]. In the 1980s, magnetic shaking was first used in magnetic shielding by Kelha et al., who observed a significant improvement in SF when the shaking field was applied to a quadrangular device [166,167]. This phenomenon is attributed to the continuous motion of the domain caused by magnetic shaking, which prevents the domain walls from freezing on a lattice defect. 

Sasada et al. applied shaking field with a frequency of 1 kHz and an amplitude of 2.9 A/m to a cylindrical device made of Metglas 2705M amorphous ribbon, which improves the SF from 3.5 to 150 [168,169,170]. Subsequently, Sasada et al. analyzed and tested the noise characteristics of a Co-based amorphous material under magnetic shaking [171]. In 2002, Nagashima et al. designed a double-layer cylindrical magnetic shield using two types of amorphous ribbon. Under the effect of magnetic shaking, the outer layer of Metglas 2705M reached a higher permeability of 5 × 10^5^ than the permalloy commonly used, and the inner layer of Metglas 2714A also achieved a permeability of 10^4^. The axial and radial shielding factors of this cylindrical magnetic shield are 700 and 20,000, respectively, but the total weight is only 1.3 kg, which is 1/4 of the equivalent permalloy device [37]. Based on these studies, Tashiro et al. investigated the effects of magnetic shaking on the incremental permeability of silicon steel, and its initial permeability increased by 45% [41]. However, the shaking field itself can be introduced into the magnetic-shielding device as interference, complicating the nearly zero-field environment, which is the main obstacle to the application of magnetic shielding.

Magnetic shaking can bring the magnetization curve closer to the anhysteretic magnetization curve with a higher initial permeability by suppressing the hysteretic losses in the magnetization process. To further characterize the effect, Yang et al. proposed a theoretical model by introducing the attenuation coefficient *S_hy_* to the terms of the hysteresis loss in the Jiles–Atherton model [42], which can be expressed as follows:

(19) $$\mu_{0}\int MdH_{e}=\mu_{0}\int M_{an}dH_{e}-S_{hy}\left[\left(1-c\right)\mu_{0}k\delta \int \frac{dM_{irr}}{dH_{e}}dH_{e}\right]$$

**Figure 12:**
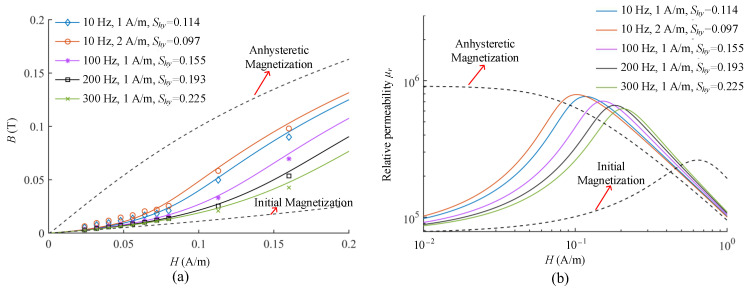
Magnetic properties under magnetic shaking with different frequencies and amplitudes. (**a**) Magnetization curves. (**b**) Relative permeabilities [42].

The magnetic properties of permalloy, under magnetic shaking with different parameters, were tested and calculated as shown in Figure 12. To minimize introducing additional interference, the magnetic shaking was only applied to the outermost permalloy layer of the three-layers MSR, which nearly doubles the shielding performance. Allmendinger et al. also applied the magnetic shaking with low frequency of 0.2 Hz and low current of 1 A to the outermost high-permeability layer of the MSR, and its shielding factors were improved by nearly 4 times [172].

## 5. Magnetic Field Compensation for Magnetic Noise Suppression

Magnetic noise is an important factor to evaluate the performance of magnetic-shielding devices, which can be divided into two parts according to the source, the noise from materials or environmental field. For the large-sized MSR, it is difficult to achieve the sufficient shielding factors by increasing the number of shielding layers due to cost constraints, which results in a significant increase in the internal magnetic noise from environment, especially at low frequencies. Therefore, the compensation coils, including uniform field coils and gradient field coils mainly, can be designed to generate an inverse field to actively suppress the internal magnetic noise. In this chapter, the design methods of compensation coil are illustrated, especially the target field method, which is a popular inverse design method. This chapter also focuses on the calculation methods regarding the coupling effect between the compensation field and ferromagnetic boundary and introduced the application of active compensation in the MCG and MEG.

### 5.1. Design Method of Compensation Coil

#### 5.1.1. Forward Design Method

In the forward design method of the coil, some elements with regular geometry are combined together as the basic structure. Based on the Biot–Savart Law, the magnetic field generated by the coil can be expressed, and the geometric parameters of the coil can be optimized to obtain the desired configuration. In 1984, Roméo et al. used the expansion of spherical harmonic function to analyze various basic geometric structure of the coil and successfully designed the low-order gradient coil [173]. The method of harmonic analysis is widely used in the design of low-order gradient coil for MRI (Magnetic Resonance Imaging) [174]. In addition, the Taylor expansion method is another commonly used method for coil design. In this method, the magnetic field generated by the coil is firstly written as the function form of the summation of the multi-order Taylor expansion, and then, the structural parameters of the coil are determined according to the coefficients of the partial derivatives of each order [175]. This method is suitable for coils with symmetrical and regular geometry, such as Helmholtz coil and Maxwell coil, and they both satisfy the second partial derivative of Taylor expansion being zero [176]. In 2018, Wu et al. designed the nested saddle coil based on the Taylor expansion method, improving the uniformity of the radial field greatly [43].

These forward design methods can effectively suppress the low-order harmonic magnetic field by adjusting the structural parameters of the coil and can greatly improve the uniformity of the magnetic field or magnetic field gradient, especially suitable for the design of axial coils and radial saddle coils used in cylindrical magnetic shields [177,178]. However, the coil structure designed by the forward design method is too simple, and the utilization rate of the desired field space is low, which cannot meet the structural constraints of different occasions.

#### 5.1.2. Inverse Design Method

Different from the forward method, the inverse design does not need to determine the specific geometric structure. The current density on the surface of the coil is calculated based on the magnetic field distribution in the desired target area and then is discretized by winding to obtain the specific coil structure. As a typical inverse method, the target-field method (TFM) is often utilized to devise special-shaped coils according to the desired fields. This method was first proposed in 1980s by Turner et al. for designing the cylindrical gradient coil used in MRI, who calculated the current distribution according to the relationship between the current density and the desired field [179]. Then, Forbes and Crozier constructed the stream function of current density based on a 2D Fourier series to restrict the coil structure on a finite plane and designed various kinds of coils to generate the uniform and gradient fields for magnetic resonance imaging (MRI) [180]. With the superiority in designing special-shaped coils that generate highly homogeneous field, the TFM has also been used to compensate the background field and construct an extremely weak magnetic environment [181]. Holmes et al. proposed six pairs of biplanar coils to produce uniform fields and gradients field for nulling background field interference, providing a low-noise zero-field environment for MEG experiment [46]. Wang et al. also designed the radial and axial uniform field coils to compensate the residual field in a cylindrical magnetic shield for an atomic magnetometer [182].

The design process of the TFM is introduced in the following, taking the biplanar coils as an instance. The coordinates of the two planes of the coils are set to (x, y, +D) and (x, y, −D), respectively. A cubic target field region with side length L is set at the center of the origin, and the structure of the biplanar coil is solved by inverse calculation through the desired field set in the region. The detailed design process is as follows:

(a) Solving the stream function and current density

The current distribution on the biplanar coil is regarded as a two-dimensional continuous fluid, which satisfies the continuity equation and the streamline differential equation:

(20) $$\nabla \cdot \vec{J}=0$$

(21) $$dr\times \vec{J}\left(r\right)=0$$

The stream function is set to *S*(*x,y*), and its differential equation should also satisfy the streamline differential equation, which can be expressed as follows:

(22) $$dS(x,y)=\frac{\delta S}{\delta x}dx+\frac{\delta S}{\delta y}dy=-J_{y}dx+J_{x}dy$$

(23) $$\left\{\begin{array}{l} J_{x}=\frac{\delta S}{\delta y}\\ J_{y}=-\frac{\delta S}{\delta x} \end{array}\right.$$

Since the current distribution is perpendicular to the *z*-axis direction, the current density in the *z*-axis direction is zero. Two-dimensional Fourier series can be used as the basis function to represent the stream function as follows [38]:

(24) $$\begin{array}{r} S(x,y)=\sum_{m=1}^{M}\left[\alpha_{m}\cos\left(\frac{\pi }{2}\left(2m-1\right)\frac{x}{L}\right)+\beta_{m}\sin\left(\frac{\pi mx}{L}\right)\right]\\ \times \sum_{n=1}^{N}\left[\gamma_{n}\cos\left(\frac{\pi }{2}\left(2n-1\right)\frac{y}{L}\right)+\delta_{n}\sin\left(\frac{\pi ny}{L}\right)\right] \end{array}$$

where $\alpha_{m}$, $\beta_{m}$, $\gamma_{n}$, and $\delta_{n}$ can be solved to determine the detail structure of the biplanar coil. The direction of the uniform field or gradient field generated by the coil is determined by the symmetry of the current density. The stream function can be further simplified according to the symmetry and the characteristic that the current density is zero at the edge of the coil surface. The symmetries and stream functions of the biplanar coils on different directions are list in Table 6 [183,184]. 

In addition to the usual target fields of homogenization and first-order gradient fields, Yuan et al. also design the biplanar coils for spatial nonlinear magnetic fields based on the above-mentioned stream functions [185]. The expression of current density can be obtained by substituting the stream function into Equation (23). Based on the Biot–Savart law, the relationship between the current density and target field can be written as follows:

(25) $$B_{t\arg et}\left(\vec{r}\right)=\frac{\mu_{0}}{2\pi }\int \frac{J(\vec{r^{\prime }})d\upsilon \times \left(\vec{r}-\vec{r^{\prime }}\right)}{{\left\|\vec{r}-\vec{r^{\prime }}\right\|}^{3}}=\sum_{m=1}^{M}\sum_{n=1}^{N}P_{mn}U_{mn}$$

where *B_target_* is the distribution function of the target field, dυ denotes the integration unit in the coil planes, $\vec{r}$ and $\vec{r^{\prime }}$ represent the position vector of the field point and the source point, and $\left\|\vec{r}-\vec{r^{\prime }}\right\|$ is the distance between the field point and the source point. However, this integral equation belongs to the Fredholm integral equation of the first kind, which cannot be solved directly.

(b) Regularization solution and stream function discretization

To solve the above integral equation, the target field is uniformly discretized into *NUM* points, so that the integral equation is transformed into the form of equation set, which is still an overdetermined system. The Tikhonov regularization, as the most commonly used regularization method of ill-posed problems, can be utilized to solve this overdetermined system by introducing a regularization matrix into the least squares linear regression, which loses the unbiasedness for high computational stability. The Tikhonov functional is formed by introducing the penalty function, such as the curvature or power loss of coil, to the least squares form of the Equation set, which can be expressed as:

(26) $$E={\left\|B(x_{num},y_{num},z_{num})-B_{target}\right\|}_{2}^{2}+\lambda {\left\|\Gamma P_{mn}\right\|}_{2}^{2}$$

where Γ is the Tikhonov regularization matrix, and *λ* is the regularization coefficient. Taking the curvature of coil as the penalty function, the Tikhonov regularization matrix can be derived as follows:

(27) $${\left\|\Gamma P_{mn}\right\|}_{2}^{2}={\iint \left\|\Delta S\right\|}_{2}^{2}d\upsilon$$

Since the Tikhonov functional has a unique minimum, the undetermined parameters *P_mn_* can be solved by setting its derivative to zero, which can be written as follows:

(28) $$x={\left(A^{T}A+\lambda \Gamma^{T}\Gamma \right)}^{-1}A^{T}B$$

where **B** represents the distribution of target field, and the matrices of **A** and **x** are as follows:

(29) $$A={\left[\begin{array}{cccc} U_{11}^{num=1} & U_{12}^{num=1} & \cdots & U_{MN}^{num=1}\\ U_{11}^{num=2} & U_{12}^{num=2} & \cdots & U_{MN}^{num=2}\\ \vdots & \vdots & \ddots & \vdots \\ U_{11}^{num=NUM} & U_{12}^{num=NUM} & \cdots & U_{MN}^{num=NUM} \end{array}\right]}_{NUM\times MN}$$

(30) $$x={\left[\begin{array}{c} P_{11}\\ P_{12}\\ \vdots \\ P_{mn} \end{array}\right]}_{MN\times 1}$$

**Figure 13:**
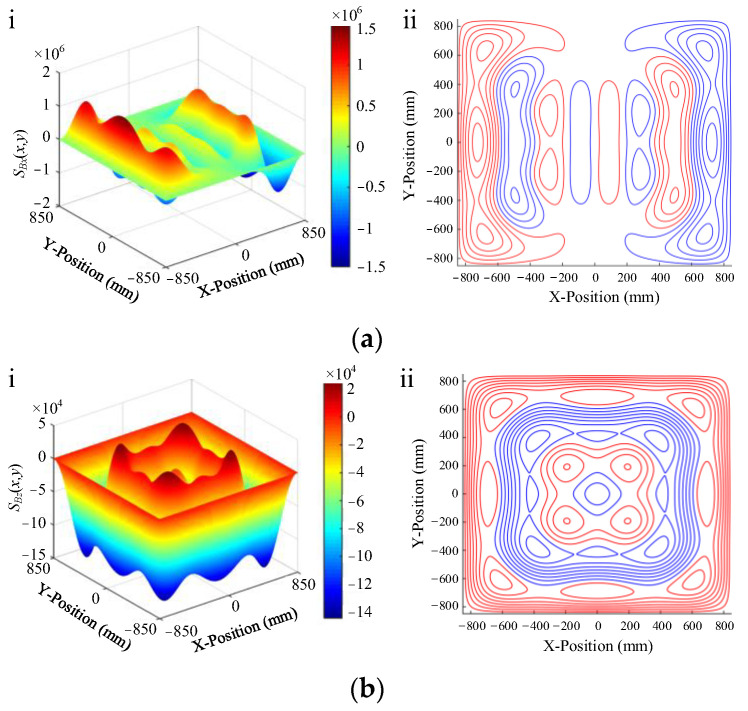
Stream functions (**i**) and coil structure (**ii**) of biplanar coils designed by TFM: (**a**) *B*_x_ coil and (**b**) *B*_z_ coil [183].

To further obtain the coil configuration, the stream function of the current distribution can be discretized using contours, as shown in Figure 13. After discretization, the direction of the current is not judged by the positive or negative of the contours but determined by the sequence of the contour coordinates, which can be further calculated by using Stokes formula. Too few contours lead to an increase in the error between the actual field distribution and the designed vale, while too many lead to a dense distribution of the coil, which is difficult to process. To avoid the error introduced by the discretization of TFM, Wang et al. utilized dedicated spiral functions to directly obtain the discrete current density distribution in the target space to design the cylindrical gradient coil [186]. Zhou et al. proposed a quasi-elliptic function fitting (EFF) method combined with particle swarm optimization (PSO) for simplifying the coil structure and improve the uniformity of field [187].

(c) Optimization of regularization coefficient

The regularization coefficient of *λ* directly determined the weight of the penalty function in the TFM. Taking the penalty function of curvature as an example, a larger coefficient reduces the overall curvature of the coil structure, thus making the coil shape tend to be simpler and increasing the error of desired field. On the contrary, a smaller coefficient makes the shape of the coil tend to be complex, resulting in increasing the difficulty of machining, which also causes a decrease in the uniformity of field. Therefore, it is necessary to optimize the regularization coefficient in an appropriate range to ensure that the coil can be machined while improving the uniformity performance. On this basis, Wang et al. proposed a hybrid optimal design of biplanar coils combined with TFM and PSO algorithm to improve the uniformities of fields or gradients, in which the undetermined parameters of stream function are directly optimized instead of the regularization coefficient [188].

**Figure 14:**
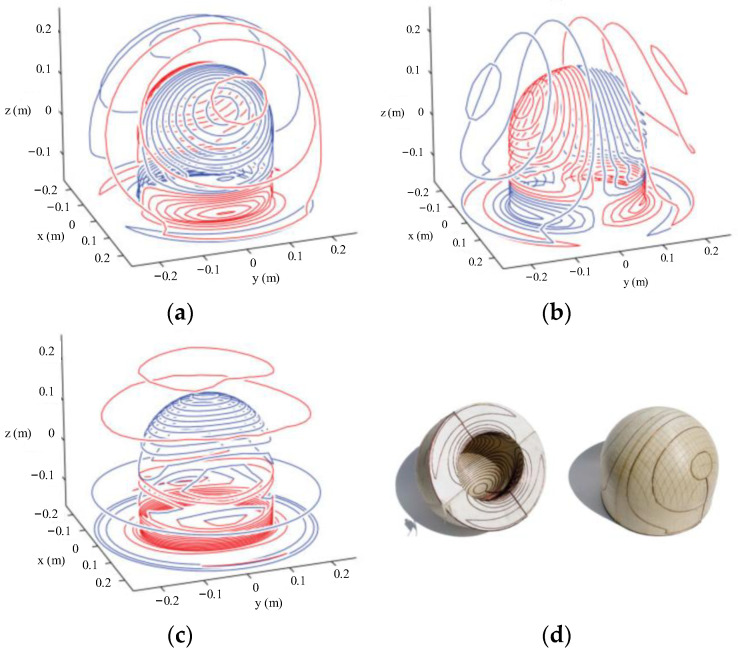
Irregular gradient coil designed by boundary element method: (**a**) d*B_x_*/d*x* coil; (**b**) d*B_y_*/d*y* coil; (**c**) d*B_z_*/d*z* coil; (**d**) gradient coil diagram [190].

The TFM is mainly suitable for the design of coil structure on the surface of regular shape, such as plane, sphere, and cylinder. In order to complete the coil design on any curved surface, a series of design methods based on finite element method (FEM) were presented [189]. Poole et al. designed the field gradient coils with a highly asymmetric headlike shape based on the inverse boundary element method, which is free from all symmetry constraints, as shown in Figure 14 [190]. The red and blue lines of the coils represent opposite currents. Zhu et al. [191] proposed the finite difference method for gradient coil design, which is appropriate for a 2D or 3D irregular shape surface. Lopez et al. proposed the design method for the gradient coils wound arbitrary surface shapes of MRI based on the equivalent relationship between the magnetized volume surrounded by a conducting surface and the surface current density [192].

### 5.2. Coupling Effect Between Compensation Field and Magnetic Shield

The compensation field generated by the coils can be significantly distorted by the high-permeability material of the magnetic shield, resulting in the reduction in the field uniformity. On the contrary, the magnetization state of the shielding layer is also affected by the compensation field, which leads to the degradation of the shielding performance. Therefore, the coupling effect between the compensation field and magnetic shield should be furthered analyzed and calculated in the coil design.

#### 5.2.1. External Compensation

**Figure 15:**
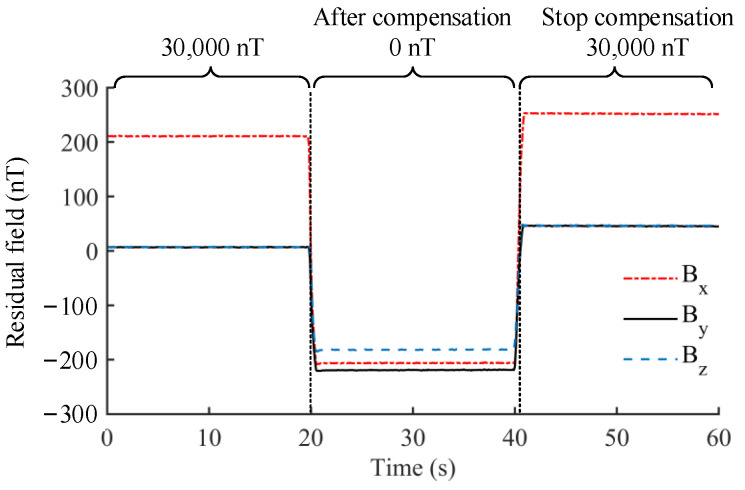
The change in residual field before and after compensation [147].

The external compensation coils are usually installed around the magnetic-shielding device for nulling the geomagnetic and environmental fields using the magnetometers placed outside the device as feedback. However, the large change in the environmental field, especially in the static field component, can directly destroy the magnetism equilibrium of the shielding material, and results in the significant increase in the residual field inside the shielding device. Yang et al. simulated the external compensation process using the cubic shielding device with the rectangular Helmholtz coil inside the MSR [147]. After compensation, the static residual field in the degaussed cubic device increased in the inverse direction, as shown in Figure 15. The residual fields of *B_x_*, *B_y_,* and *B_z_* were changed from 210 nT, 6 nT, and 7 nT to −206 nT, −220 nT, and −181 nT, respectively, under the influence of the magnetic compensation. It demonstrated that the magnetism equilibrium between the shielding material and the environmental field is broken due to the external compensation, and the new equilibrium can be reconstructed by degaussing after the compensation.

**Figure 16:**
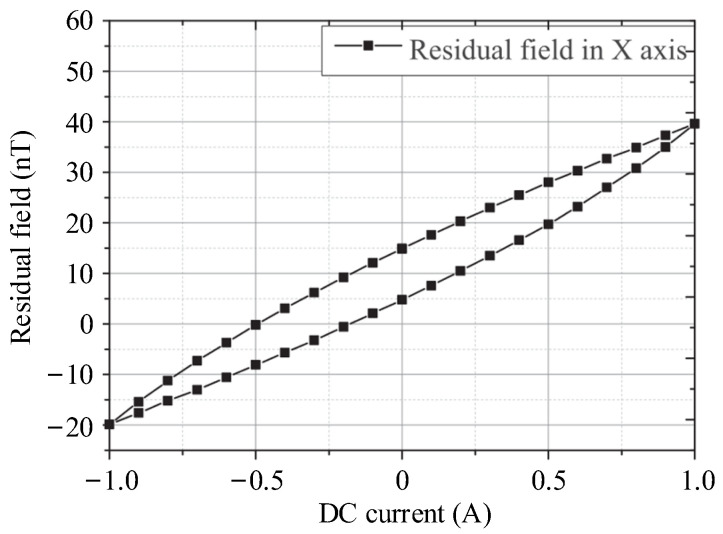
Relationship between the internal residual field and the current in the external coils [193].

Another method of external compensation is that the internal field, rather than the environmental field, is indirectly compensated based on the reference sensors inside the device through the external coils. The compensation field from the external coil first causes the change in the magnetization state of the shielding material and then affects the internal magnetic field of the device. This compensation process is nonlinear and time-delayed due to the hysteresis property of the material [193]. As shown in Figure 16, the magnetic field inside magnetic shield changes similarly to the hysteresis loop when a sinusoidal magnetic field with low frequency is applied through the external coil. In order to address the limitation of the coupling characteristics, Li et al. established an active magnetic compensation system based on the model-free adaptive control method with a radial basis function neural network to realize the high-precision compensation in the MSR [194].

#### 5.2.2. Internal Compensation

Compared with the external coils, the advantage of the internal compensation is that the compensation field in the opposite direction can be generated for suppressing the residual field and magnetic noise inside magnetic shield directly. However, the compensation field generated by the internal coils is inevitably distorted due to the effect of the high-permeability shielding layers, which results in the degradation of the compensation effectiveness. To solve this problem, there are two main solutions. One is to consider the coupling effect in the coil design process, the other is to design self-shielding coil.

For the cylindrical magnetic shield with infinite length, the coupling effect can be deduced theoretically. The distribution of magnetic vector potential in air and shielding layer satisfies the Laplace equation and Poisson equation, respectively. Bidinosti et al. provided a general solution for the coupling effect of an infinitely long and hollow cylinder with uniform linear permeability based on the spatial Fourier components of the applied surface current [195]. Taking the single-layer cylindrical magnetic shield as an instance, the field distribution is affected by the three concentric surface currents, including internal coil (*R = a*), inner, and outer surface of the layer (*R = b & R = c*), which can be expressed as follows:

(31) $$\begin{array}{l} \left(\begin{array}{l} B_{\rho }\\ B_{\phi }\\ B_{z} \end{array}\right)=\frac{-\mu_{0}}{2\pi }\sum_{m=-\infty }^{\infty }e^{im\phi }\int_{-\infty }^{\infty }dke^{ikz}\\ \times \left(\begin{array}{l} \frac{k}{i}\left[aP_{m}\left(a\right)F_{\phi }^{m}\left(k\right)+bP_{m}(b)f_{b\phi }^{m}\left(k\right)+cP_{m}\left(c\right)f_{c\phi }^{m}\left(k\right)\right]\\ \frac{m}{\rho }\left[aQ_{m}\left(a\right)F_{\phi }^{m}\left(k\right)+bQ_{m}(b)f_{b\phi }^{m}\left(k\right)+cQ_{m}\left(c\right)f_{c\phi }^{m}\left(k\right)\right]\\ k\left[aQ_{m}\left(a\right)F_{\phi }^{m}\left(k\right)+bQ_{m}(b)f_{b\phi }^{m}\left(k\right)+cQ_{m}\left(c\right)f_{c\phi }^{m}\left(k\right)\right] \end{array}\right) \end{array}$$

(32) $$Q_{m}\left(s\right)=\left\{\begin{array}{l} I_{m}\left(k\rho \right){K^{\prime }}_{m}\left(ks\right),\;\rho <s\\ {I^{\prime }}_{m}\left(ks\right)K_{m}\left(k\rho \right),\rho >s\; \end{array}\right.$$

where r(ρ,ϕ,z) and $\;r^{’}(a,\phi^{’},z^{’})$ are the field point and source point; *b* and *c* represent the inner and outer diameter of the shielding layer; $F_{\phi }^{m}(k)$, $f_{b\phi }^{m}(k)$, and $f_{c\phi }^{m}(k)$ are the *m*th order Fourier components of the azimuthal currents on each surface; *I_m_* and *K_m_* denote the modified Bessel function of the first and second kind, respectively. On this basis, Zhao et al. proposed an analytical model to calculate the coupling effect of the enclosed cylindrical magnetic shield [196] and designed the axial uniform coils for the compensation of internal residual field [197]. Packer et al. introduced the analytical model of the interaction between an arbitrary static current flow on a cylinder and enclosed cylindrical magnetic shield into the inverse method of coil design [198].

**Figure 17:**
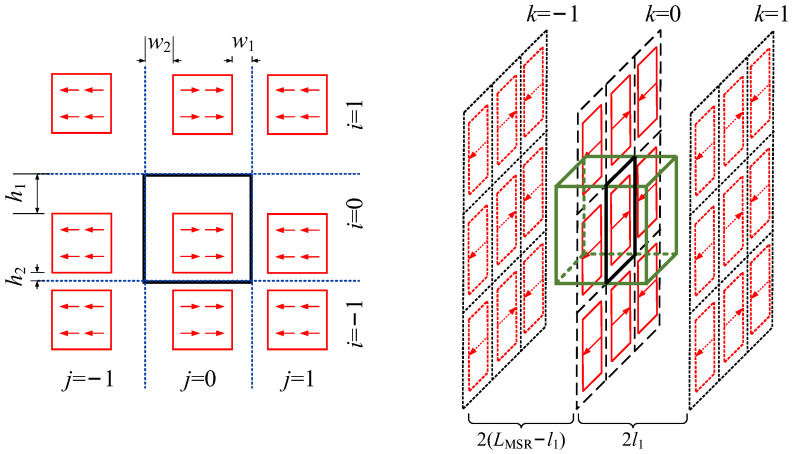
Equivalent coil array based on image method [183].

For the cubic magnetic-shielding device, such as MSR, the coupling effect cannot be calculated theoretically due to the cubic boundary conditions. The image method, as a common method for electromagnetic field analysis, is appropriate for the magnetic field analysis of planar medium with high permeability. By this method, the infinite surface with high permeability is equivalent to a mirror to reflect the surrounding magnetic source and other magnetic medium. Therefore, the six planes of cubic shielding device reflect each other and are regarded as infinite surfaces. Based on the image method, the internal coils can be equivalent to the coil array as shown in Figure 17 [183]. In the forward design method, the image method is often utilized to calculate the coupling effect of magnetic shield for the optimization of coil parameters. Pan et al. optimized the structure of the Merritt coil based on the image method to avoid the influence of the permalloy layers and improved the field uniformity by 63% in MSR [48]. Liu et al. designed a built-in coil system consisting of four identical square windings attached to the inside walls of the Berlin Magnetically Shielded Room (BMSR-2) [19] for ultra-low field spin precession experiment, and the spacings of these windings were optimized using the image method to enhance the field homogeneity [199]. Jin et al. combined the multi-reflection theory on the basis of the traditional image method and further considered the influence of the thickness of the high-permeability shielding layer on the coupling effect, which improved the calculation accuracy [200]. In the inverse design method, the image method is directly introduced into the relationship between the current density and target field. Han et al. proposed the complete design flow of the biplanar coil inside MSR based on the combination of the TFM and image method and designed the magnetic compensation system for nulling the background field in MEG experiments, including the uniform field and gradient coils [184].

The self-shielding coil can generate the desired field distribution, and the external magnetic field decays rapidly to avoid the coupling effect with the high-permeability ferromagnetic boundary. The self-shielding coil is commonly utilized in MRI devices as a gradient coil to prevent the eddy currents caused by the alternating fields in the outer metal layer [180,201]. Wu et al. proposed a self-shielding coil system consisting of pairs of circular coils distributed on two coaxial cylinder surfaces using the Taylor expansion method to ensure the uniformity of the internal field and using the TFM to ensure the attenuation of the external field [202]. On this basis, Wang et al. optimized the self-shielding coil system by a modified pigeon-inspired algorithm for miniature atomic sensors [203]. Chen et al. introduced the self-shielding constraint into the TFM and designed two sets of biplane coils for achieving uniform magnetic field in the target area and near-zero field near the shielding layer simultaneously, which has higher coupling suppression ability and is more suitable for irregular walls [75].

### 5.3. Suppression of Magnetic Noise and Motion Artifact

**Figure 18:**
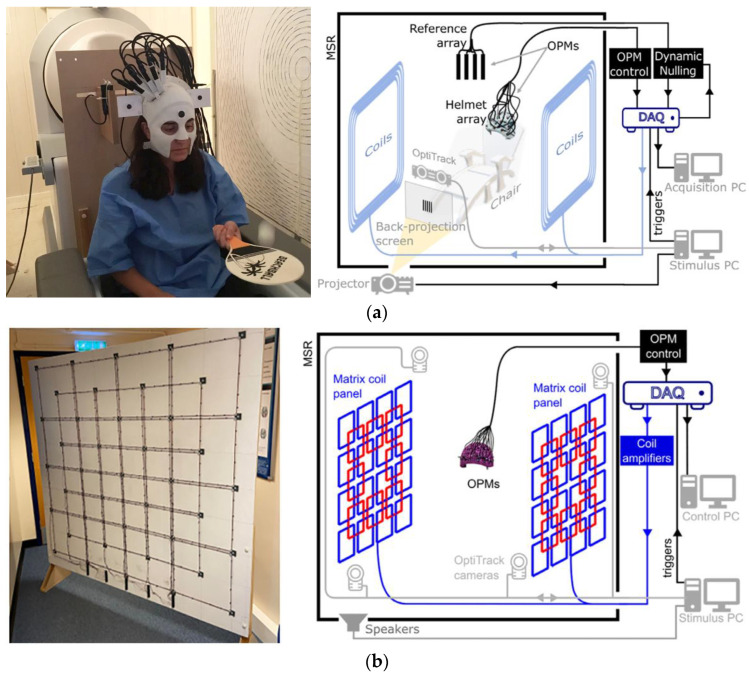
Compensation coils system in OPM–MEG: (**a**) biplanar coils and (**b**) matrix coils [15,204,205].

**Figure 19:**
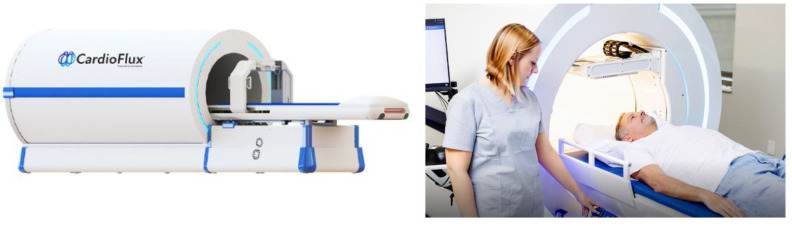
Cylindrical magnetic shield with single end opening for OPM–MCG [208].

The magnetic noise introduced by the environment in the magnetic shield is a crucial factor affecting the measurement of extremely weak magnetic signals, especially the biological magnetic field in the low-frequency band. Taking the MEG as an instance, the extracranial magnetic inductions are typically measured on a scale of 100 fT, and the frequency content of its neuromagnetic field mostly lies in a band from 1 to 80 Hz. However, the geomagnetic noise within low-frequency bands is difficult to meet the requirements of the MEG measurement only by passive magnetic shields. Holmes et al. established the compensation system including six pairs of biplanar coils designed by TFM and four atomic magnetometers for nulling the background field in MEG experiment [38,46]. The largest components of the static field and gradient were reduced by 46 and 13 times, respectively, and the low-frequency noise at 0.01 Hz was suppressed with a factor of 40 dB. Later, on this basis, the MEG helmet was tracked for recording the participants’ head motion, and the artifacts caused by the movement were reduced significantly through the compensation system of biplanar coils [204], as shown in Figure 18a. In addition, Holmes et al. proposed a compensation method based on the matrix coils, which is composed of 48 square unit coils on two planes, as shown in Figure 18b. In this way, the compensation field can be flexibly adjusted in the target region, and the noise induced by participant movement was canceled with low latency [205,206]. The cardiac magnetic signal is on the order of 10 pT, much higher than the neuromagnetic field of brain, and the cylindrical magnetic shield with single end opening is often used to provide the nearly zero-field environment for more convenient MCG measurement [207,208], as shown in Figure 19. In order to improve the anti-disturbance ability of the MCG measurement, Zhang et al. adopted the extended state observer (ESO) to estimate the magnetic noise and established the disturbance feedforward control loop based on the compensation system composed of the radial and saddle coils [209].

## 6. Conclusions and Prospects

In this paper, the recent advances in the enhancement methods of magnetic shielding performance have been reviewed from the perspective of low-noise materials, magnetization control, and active compensation. These methods offer more focused and cost-effective approaches, as opposed to simply increasing the number of masking layers to enhance performance. Using a low-noise soft magnetic material as the innermost layer of multi-layer shielding device can effectively suppress the interference from shielding material noise, such as that the magnetic measurement sensitivity of 160 at/Hz^1/2^ were achieved based on the ferrite magnetic shield [11]. The magnetization control methods of degaussing and magnetic shaking further explore the limit of magnetic properties by adjusting the magnetization state of shielding materials and reduce the static field and gradient inside magnetic shield. For instance, an unprecedented residual static field of <130 pT was achieved over 0.5 × 0.5 × 0.5 m^3^ inside the MSR [40]. The active compensation based on coils can directly suppress the magnetic noise from the external environment, and further extend the nearly zero-field space inside the magnetic shield, which is widely used in weak magnetic imaging, such as MEG [15]. In addition, these methods can also be regarded as means of upgrading the completed shielding device for achieving high performance at low cost. Some unresolved issues and challenges still need to investigated more deeply, such as the following:

(1) There is a clear gap between the permeability of low magnetic noise materials and common soft magnetic materials, such as permalloy. Deeper optimizations of composition, process techniques, and nanostructuring are needed to further enhance the magnetic performance, enabling more effective shielding against strong magnetic fields. Furthermore, traditional ferrites and nanocrystalline materials mainly shield magnetic fields at relatively low frequencies. The weak shielding performance of broader frequency ranges restricts the further application of the low magnetic noise materials.
(2) The practical magnetization state is significantly lower than the theoretical limit described by the anhysteretic magnetization curve. Moreover, there is currently no effective method for characterizing the magnetization state of the entire magnetic-shielding device after degaussing. Further optimize the degaussing process, including its waveform and coil, to reduce the loss during the process of magnetic equilibration.
(3) The suppression effect of the magnetic shaking field on the hysteretic loss in magnetization is lack of theoretical characterization microscopically, and its efficiency needs to be improved to further approach the anhysteretic state. In addition, the shaking field, as a noise source, complicates the internal environment of magnetic shield in practical applications, which also needs to explore better solutions.
(4) For magnetic compensation systems of magnetic shield, the improvement of the uniformity of magnetic field generated by coil is one of the research focuses currently. However, the problems of small scale, narrow bandwidth, and linear field distribution deserve more attention and research. Furthermore, the magnetic compensation system suitable for MCG and MEG during motion and the corresponding motion artifact suppression methods need to be further explored.

## Figures and Tables

**Table 1:** Calculation formulas for low-frequency magnetic-shielding factors of various shapes.

Shapes of Magnetic Shields	Calculation Formula	Annotation	Refs.
Single-layer spherical shape (Shield thickness can be ignored)	$SF\approx 1+\frac{2\mu_{r}}{3R_{1}}d$	*μ_r_* is the relative permeability; *R*_1_ is the outer radii.	[59]
Single-layer spherical shape (Shield thickness cannot be ignored)	$SF=1+\frac{2{\left(\mu_{r}-1\right)}^{2}(R_{1}^{3}-R_{2}^{3})}{9\mu_{r}R_{1}^{3}}$	*μ_r_* is the relative permeability, *R*_1_ is the outer radii, and *R*_2_ is the inner radii.	[60]
Single-layer, large-scale, open-ended cylindrical shape	$SF_{T}=\frac{\mu_{r}t}{2R}$ $SF_{A}\approx \frac{2\mu_{r}t\sqrt{R}}{L^{3/2}}$	*SF_T_* and *SF_A_* are the transverse and axial shielding factors, *μ_r_* is the relative permeability, *R* is the radius, *L* is the length, and t is the thickness.	[61]
Single-layer cubic shape	$SF\approx 1+0.8\frac{\mu_{r}}{L}d$	*μ_r_* is the relative permeability; *d* and *L* are the thickness and length of the shielding wall.	[60]
Double shells	$SF\approx 1+SF_{1}+SF_{2}+SF_{1}SF_{2}(1-\frac{D_{i}^{3}}{D_{o}^{3}})$	*SF*_1_ and *SF*_2_ are the shielding factors of one-walled shields; *D*_i_ and *D*_o_ are the inside and outside diameters of the space between the two shells.	[62]

**Table 2:** Summary of magnetic-shielding devices performances.

Time	Country	Magnetic-Shielding Devices	Shape	Dimensions	Structure	Performance	Refs.
1980	Germany	BMSR(The Berlin Magnetically Shielded Room)	Cubic	External dimensions of 4.6 m × 4.6 m × 4.6 m and internal dimensions of 2.25 m × 2.25 m × 2.25 m	Seven shells including a single layer of 15 mm thick copper and six layers of permalloy	SF(@0.1 Hz) = 1 × 10^4^	[63,64,65]
1996	Japan	COSMOS	Quasi spherical ‘soccer’ shape	Outer diameter of 6 m and inner diameter of 4 m	Four layers of permalloy and one layer of aluminum	SF(@1 Hz) = 4.2 × 10^5^	[58,66]
2000	Germany	BMSR-2	Cubic	Internal dimensions of 2.9 m × 2.9 m × 2.9 m	A total of nine sandwich layers supporting the floor, seven layers of MUMETALL (iron-nickel-copper-manganese alloy) with varying thickness and one layer aluminum with thickness of 10mm	SF(@0.01 Hz) = 7.5 × 10^4^; SF(@0.01 Hz) = 2 × 10^6^ with active shielding	[19]
2002	America	Six-layer magnetically shielded room	Cubic	External dimensions of 5.3 m × 4.3 m × 4.1 m and internal dimensions of 4.0 m × 3.0 m × 2.4 m	Three nested shells, each with a layer of pure aluminum and a high-permeability ferromagnetic layer	SF(@0.01 Hz) = 1630	[57]
2011	America	NSE-MSR(magnetically shielded room for the neutron spin echo spectrometer)	Cubic	Internal dimensions of 17 m × 5.5 m × 4.4 m	Two layers of high-permeability materials and a 300mm thick high-density concrete layer in between for radiation shielding	SF(low frequency) = 100; customized for neutron spin echo spectrometers	[67].
2014	Germany	TUM magnetically shielded room	Cubic	External dimensions of 2.50 m × 2.78 m × 2.30 m, internal dimensions of 1.90 m × 2.78 m × 1.90 m	Two layers of μ-metal and one layer of 8mm thick aluminum	A residual field of (700 ± 200) pT within a central volume of 1m^3^ and a field gradient less than 300 pT/m	[18]
2021	China	BUAA MSR (for MEG test)	Cubic	External dimensions of 4.5 m × 3.5 m × 3.0 m, internal dimensions of 4.1 m × 3.2 m × 2.8 m	Three layers of 1J85 permalloy and one layer of 10mm thick aluminum	SF(@0.01 Hz) = 210.5; SF(@0.01 Hz) = 489.1 with magnetic shaking	[42]
2022	England	Lightweight magnetically shielded room	Cubic	Internal dimensions of 2.4 m × 2.4 m × 2.4 m	Two layers of μ-metal and one layer of copper with a new ‘window coil’ active shielding system composed of 27 rectangular coils	Residual magnetic field is 4.84 nT; |B| = (670 ± 160) pT combined with active shielding	[20]

**Table 3:** Magnetic field noise from high-permeability materials of symmetrical geometry.

Geometry	Magnetic Noise Due to Johnson Noise Current	Annotation	Refs.
Infinite plate	$\delta B_{curr}=\frac{1}{\sqrt{6\pi }}\frac{\mu_{0}\sqrt{kT\sigma t}}{a}$	$\mu_{0}$ is the permeability of vacuum, *k* is the Boltzman constant, *T* is the Kelvin temperature, σ is the conductivity, *t* is the thickness, and a is the position on the *z*-axis. *J_n_*(*x*) is the Bessel function of order *n*.	[31]
Spherical shell	$\delta B_{curr}=\frac{1}{\sqrt{2\pi }}\frac{\mu_{0}\sqrt{kT\sigma t}}{a}$		
Infinite cylindrical shell (axial)	$\delta B_{curr}=\sqrt{\frac{2G}{3\pi }}\frac{\mu_{0}\sqrt{kT\sigma t}}{a},\;G\approx 0.435$		
Finite, closed cylindrical shell (axial)	$F_{1}=\underset{\alpha }{\sum }\frac{1}{sinh^{2}\frac{\alpha L}{2a}}\cdot \frac{1}{J_{1}^{2}\left(\alpha \right)}$ $F_{2}=\int_{0}^{1/2}dx\frac{L}{a}{\left[\underset{\alpha }{\sum }\frac{1}{J_{1}\left(\alpha \right)}\cdot \frac{cosh\frac{\alpha Lx}{a}}{sinh\frac{\alpha L}{2a}}\right]}^{2}$ $\delta B_{curr}=\sqrt{\frac{2G}{3\pi }}\frac{\mu_{0}\sqrt{kT\sigma t}}{a},\;$ $G=F_{1}\left(\frac{L}{a}\right)+2F_{1}\left(\frac{L}{a}\right)$		
Finite, closed cylindrical shell (Longitudinal and transverse magnetic noise spectrum)	$\delta B_{hyst}^{L}(f)=\frac{2\sqrt{kT\pi \frac{\tan\delta }{\mu^{\prime }}\frac{1}{t}\beta }}{\pi \sqrt{f}AI}$ $\delta B_{hyst}^{T}(f)=\frac{\sqrt{4kT}\sqrt{\int_{V}\mu^{\prime \prime }H_{m}^{2}dV}}{\sqrt{2\pi f}AI}$ $\begin{array}{c} \beta =2{\int_{0}^{r}\rho \left[\sum_{\zeta }dq\mu_{0}\frac{J_{1}\left(\frac{\rho \zeta }{r}\right)\coth\left(\frac{L\zeta }{2r}\right)}{\pi r^{2}J_{1}{\left(\zeta \right)}^{2}}\right]}^{2}d\rho \\ +2\int_{0}^{L/2}1/r\left[\sum_{\zeta }\left(dq\mu_{0}\frac{\coth\left(\frac{L\zeta }{2r}\right)}{r\pi J_{1}\left(\zeta \right)}\right.\right.\\ {\left.\left.+2\mu_{0}dq\frac{\coth\left(\frac{L\zeta }{2r}\right)\sinh{\left[\zeta \left(\frac{L}{4r}-\frac{z}{2r}\right)\right]}^{2}}{r\pi J_{1}\left(\zeta \right)}\right)\right]}^{2}d_{z} \end{array}$	*T*$\;\mathrm{is}\;\mathrm{the}\;\mathrm{Kelvin}\;\mathrm{temperature},\;k\;\mathrm{is}\;\mathrm{the}\;\mathrm{Boltzman}\;\mathrm{constant},\;\tan\delta =\mu^{\prime \prime }/\mu^{\prime }$$\;\mathrm{is}\;\mathrm{the}\;\mathrm{loss}\;\tan\mathrm{gent},\;A\mathrm{is}\;\mathrm{the}\;\mathrm{area}\;\mathrm{of}\;\mathrm{the}\;\mathrm{excitation}\;\mathrm{coil},\;I\;\mathrm{is}\;\mathrm{the}\;\mathrm{oscillating}\;\mathrm{current}\;\mathrm{with}\;\mathrm{the}\;\mathrm{frequency}\;\mathrm{of}\;f,\;q\;\mathrm{is}\;\mathrm{charge},\;r,\;L\;\mathrm{and}\;t\;\mathrm{are}\;\mathrm{the}\;\mathrm{outer}\;\mathrm{radius},\;\mathrm{length}\;\mathrm{and}\;\mathrm{thickness}\;\mathrm{of}\;\mathrm{the}\;\mathrm{cylindrical}\;\mathrm{shell},\;\mathrm{respectively},\;(\rho ,z)\;\mathrm{are}\;\mathrm{the}\;\mathrm{coordinates}\;\mathrm{of}\;\mathrm{the}\;\rho -z\;\mathrm{plan},\;J_{s}(\zeta )\;\mathrm{is}\;\mathrm{Bessel}\;\mathrm{function}\;\mathrm{of}\;\mathrm{order}\;s,\;\mathrm{and}\;\zeta \;\mathrm{is}\;a\;\mathrm{series}\;\mathrm{of}\;\mathrm{values},\;\mathrm{where}\;J_{s}(\zeta )=0$.	[25]
Infinite, hollow conducting cylinder (longitudinal magnetic field noise on the axis of the shield)	$\delta B_{magn}=\frac{0.26\mu_{0}}{r\sqrt{t}}\sqrt{\frac{4kT\mu^{\prime \prime }}{\omega {\mu^{\prime }}^{2}}}$ $\delta B_{eddy}=\frac{\mu_{0}\sqrt{t}}{4r}\sqrt{3kT\sigma }C\left(\mu \right)$	$\mu_{0}$ is the permeability of vacuum, *r* and *t* are the inner radius and the thickness of the shield, *σ* is the conductivity, *C*(*μ*) = 1 for *μ*/*μ*_0_ = 1, and *C*(*μ*) ≈ 0.7 for *μ*/*μ*_0_ >> 1.	[77]

**Table 4:** Preparation method, initial permeability, saturation magnetization, remanent magnetization, and coercivity of Mn-Zn ferrites.

Spinel Ferrites	Preparation Method	Initial Permeability	Saturation Magnetization *M_s_*	Remanent Magnetization *M_r_*	Coercivity *H_c_*	Refs.
(Mn_0.55_Zn_0.35_Fe_0.1_)Fe_2_O_4_ ceramic	Solid reaction (sintered)	/	4960 ± 20 Gs	2.2 ± 0.6 Gs	0.8 ± 0.3 Oe	[112]
Ni_0.4_Zn_0.5_Mn_0.1_Fe_2_O_4_	Sol–gel (Citric acid)	/	68.40 emu/g	/	/	[113]
Ni_0.4_Zn_0.6_Fe_2_O_4_		/	55.44 emu/g	/	/	
Ni_0.4_Zn_0.55_Mn_0.05_Fe_2_O_4_		/	37.18 emu/g	/	/	
Ni_0.4_Zn_0.45_Mn_0.15_Fe_2_O_4_		/	62.81 emu/g	/	/	
Ni_0.4_Zn_0.4_Mn_0.2_Fe_2_O_4_		/	53.63 emu/g	/	/	
Ni_0.4_Zn_0.35_Mn_0.25_Fe_2_O_4_		/	61.76 emu/g	/	/	
ZnFe_2_O_4_	Sol–gel (Citric acid)	2596	52.35 emu/g	/	44 Oe	[106]
Zn_0.9_Mn_0.1_Fe_2_O_4_		2472	50.77 emu/g	/	132 Oe	
Zn_0.7_Mn_0.3_Fe_2_O_4_		2033	48.78 emu/g	/	174 Oe	
Zn_0.6_Mn_0.4_Fe_2_O_4_		1835	47.58 emu/g	/	218 Oe	
Zn_0.5_Mn_0.5_Fe_2_O_4_		1543	45.80 emu/g	/	262 Oe	
Mn_0.2_Zn_0.8_Fe_2_O_4_	Hydrothermal		26 emu/g	1.71 emu/g	56 Oe	[108]
Mn_0.4_Zn_0.6_Fe_2_O_4_		/	46 emu/g	4.7 emu/g	60 Oe	
Mn_0.6_Zn_0.4_Fe_2_O_4_		/	77 emu/g	9.5 emu/g	62.6 Oe	
Mn_0.8_Zn_0.2_Fe_2_O_4_		/	70 emu/g	10.1 emu/g	75.1 Oe	
MnFe_2_O_4_		/	68 emu/g	10.5 emu/g	86.7 Oe	
ZnFe_2_O_4_	One-pot microwave combustion	/	2.598 emu/g	0.014 emu/g	7.472 Oe	[114]
Mn_0.2_Zn_0.8_Fe_2_O_4_		/	5.868 emu/g	0.162 emu/g	19.65 Oe	
Mn_0.4_Zn_0.6_Fe_2_O_4_		/	23.06 emu/g	1.134 emu/g	23.72 Oe	
Mn_0.6_Zn_0.4_Fe_2_O_4_		/	36.75 emu/g	2.015 emu/g	37.42 Oe	
Mn_0.8_Zn_0.2_Fe_2_O_4_		/	48.09 emu/g	8.473 emu/g	53.93 Oe	
MnFe_2_O_4_		/	60.99 emu/g	11.48 emu/g	64.78 Oe	
Mn_0.5_Zn_0.5_Fe_2_O_4_	Chemical co-precipitation	/	23.95 emu/g	0 emu/g	0	[115]
Mn_0.5_Zn_0.5_Sm_0.1_Fe_1.9_O_4_		/	31.72 emu/g	0 emu/g	0	
Mn_0.5_Zn_0.5_Sm_0.3_Fe_1.7_O_4_		/	37.75 emu/g	3.55 emu/g	180	
Mn_0.5_Zn_0.5_Sm_0.5_Fe_1.5_O_4_		/	42.10 emu/g	8.50 emu/g	250 Gs	
Mn_0.782_Zn_0.128_Fe_0.09_^2+^ Fe_2_^3+^O_4_	Solid reaction	~2100	/	/	/	[116]
Mn_0.778_Zn_0.128_ Ti_0.004_^4+^Fe_0.098_^2+^ Fe_1.992_^3+^O_4_		~2050	/	/	/	
Mn_0.778_Zn_0.128_ Sn_0.004_^4+^Fe_0.098_^2+^ Fe_1.992_^3+^O_4_		~1900	/	/	/	
Mn_0.5_Zn_0.5_Fe_2_O_4_	Nitrate–citrate auto-combustion	/	44.32 emu/g	12.31 emu/g	70 Oe	[117]
	Nitrate–citrate auto-combustion+ annealed at 1200 °C in air	/	48.15 emu/g	8.724 emu/g	51 Oe	
	Nitrate–citrate auto-combustion+ annealed at 600 °C in air	/	56.37 emu/g	13.72 emu/g	32 Oe	
ZnFe_2_O_4_	Sol–gel auto-combustion	/	3.10 emu/g	0.50 emu/g	50 Oe	[118]
ZnFe_1.75_Cr_0.125_Al_0.125_O_4_		/	3.00 emu/g	0.60 emu/g	90 Oe	
ZnFe_1.5_Cr_0.25_Al_0.25_O_4_		/	2.75 emu/g	0.60 emu/g	60 Oe	
ZnFe_1.25_Cr_0.375_Al_0.375_O_4_		/	2.75 emu/g	0.60 emu/g	50 Oe	
ZnFe_1.0_Cr_0.5_Al_0.5_O_4_		/	2.50 emu/g	0.50 emu/g	110 Oe	
ZnFe_0.5_Cr_0.75_Al_0.75_O_4_		/	2.50 emu/g	0.50 emu/g	50 Oe	
ZnCr_1.0_Al_1.0_O_4_		/	1.90 emu/g	0.50 emu/g	50 Oe	
Ni_0.1_Zn_0.9_Fe_2_O_4_	Chemical co-precipitation	/	23.95 emu/g	0 emu/g	0	[119]
Ni_0.3_Zn_0.7_Fe_2_O_4_		/	31.72 emu/g	0 emu/g	0	
Ni_0.5_Zn_0.5_Fe_2_O_4_		/	37.75 emu/g	3.55 emu/g	200 Gs	
Ni_0.4_Zn_0.6_Fe_2_O_4_	Sol–gel (Citric acid)	929	65.98 emu/g	1.25 emu/g	19.34 Oe	[107]
Ni_0.4_Zn_0.55_Mn_0.05_Fe_2_O_4_		188	88.50 emu/g	1.77 emu/g	27.60 Oe	
Ni_0.4_Zn_0.5_Mn_0.1_Fe_2_O_4_		159	69.52 emu/g	1.67 emu/g	19.60 Oe	
Ni_0.4_Zn_0.45_Mn_0.15_Fe_2_O_4_		206	71.99 emu/g	1.22 emu/g	21.24 Oe	
Ni_0.4_Zn_0.4_Mn_0.2_Fe_2_O_4_		122	70.44 emu/g	1.41 emu/g	20.59 Oe	
Ni_0.4_Zn_0.35_Mn_0.25_Fe_2_O_4_		218	72.80 emu/g	1.53 emu/g	20.64 Oe	
Ni_0.4_Zn_0.55_Co_0.05_Fe_2_O_4_		411	60.77 emu/g	1.09 emu/g	19.73 Oe	
Ni_0.4_Zn_0.5_Co_0.1_Fe_2_O_4_		325	72.52 emu/g	1.45 emu/g	21.25 Oe	
Ni_0.4_Zn_0.45_Co_0.15_Fe_2_O_4_		72	74.16 emu/g	1.33 emu/g	23.59 Oe	
Ni_0.4_Zn_0.4_Co_0.2_Fe_2_O_4_		36	65.65 emu/g	2.04 emu/g	18.42 Oe	
Ni_0.4_Zn_0.35_Co_0.25_Fe_2_O_4_		27	91.15 emu/g	2.10 emu/g	33.36 Oe	

**Table 5:** Composition, saturation magnetization, coercivity, and effective permeability of amorphous and nanocrystalline.

Type	Composition	*B_s_*	*H_c_*	*μ*_e_ (1 kHz)	Core Loss (kW/m^3^)	Refs.
Finemet	Fe_73.5_Cu_1_Nb_3_Si_13.5_B_9_	1.24 T	0.53 A/m	100,000	280	[133]
	Fe_73.5_Cu_1_Nb_3_Si_16.5_B_6_	1.18 T	1.1 A/m	75,000	280	[133]
	Fe_80_Si_7_B_9_Nb_3_Cu_1_	1.57 T (@10kHz)	26.3 A/m(@10kHz)	8700 (@10kHz)	124 (100 kHz, 0.1T)	[141]
Nanoperm	Fe_91_Zr_7_B_2_	1.70 T	7.2 A/m	14,000	/	[134]
	Fe_86_Zr_7_B_6_Cu_1_	1.52 T	3.2 A/m	48,000	/	[134]
Hitperm	(Fe_0.65_Co_0.35_)_83_Si_4_Cu_1_B_8_P_4_	1.68T	5.4 A/m	29,000		[142]
Nanomet	Fe_85_Si_2_B_8_P_4_Cu_1_	1.85 T	5.8 A/m	27,000	/	[143]
	Fe_83.3_Si_4_B_8_P_4_Cu_0.7_	1.88 T	7 A/m	25,000	/	[144]
Amorphous Alloy	Fe-Si-B-M	1.41 T	6.9 A/m	6000	460	[133]
	Co-Fe-Si-B-M	0.53 T	0.32 A/m	80,000	300	[133]

**Table 6:** Symmetries and stream functions of the biplanar coils.

Type of Coils	X Symmetry	Y Symmetry	Z Symmetry	Stream Function
*B_x_*	A/S	S	A/S	$S(x,y)=\sum_{m=1}^{M}\sum_{n=1}^{N}P_{mn}\sin(\frac{m}{L}\pi x)\cos(\frac{2n-1}{2L}\pi y)$
d*B_x_/*d*z*	A/S	S	S	
*B_y_*	S	A/S	A/S	$S(x,y)=\sum_{m=1}^{M}\sum_{n=1}^{N}P_{mn}\cos(\frac{2m-1}{2L}\pi x)\sin(\frac{n}{L}\pi y)$
d*B_y_/*d*z*	S	A/S	S	
*B_z_*	S	S	S	$S(x,y)=\sum_{m=1}^{M}\sum_{n=1}^{N}P_{mn}\cos(\frac{2m-1}{2L}\pi x)\cos(\frac{2n-1}{2L}\pi y)$
d*B_z_/*d*z*	S	S	A/S	
d*B_x_/*d*y*	A/S	A/S	A/S	$S(x,y)=\sum_{m=1}^{M}\sum_{n=1}^{N}P_{mn}\sin(\frac{m}{L}\pi x)\sin(\frac{n}{L}\pi y)$

## Data Availability

The original contributions presented in the study are included in the article, further inquiries can be directed to the corresponding authors.
